# Just-in-Time Adaptive Mechanisms of Popular Mobile Apps for Individuals With Depression: Systematic App Search and Literature Review

**DOI:** 10.2196/29412

**Published:** 2021-09-28

**Authors:** Gisbert W Teepe, Ashish Da Fonseca, Birgit Kleim, Nicholas C Jacobson, Alicia Salamanca Sanabria, Lorainne Tudor Car, Elgar Fleisch, Tobias Kowatsch

**Affiliations:** 1 Centre for Digital Health Interventions Department of Management, Technology, and Economics ETH Zurich Zurich Switzerland; 2 Centre for Digital Health Interventions Institute of Technology Management University of St. Gallen St. Gallen Switzerland; 3 Experimental Psychopathology and Psychotherapy Department of Psychology University of Zurich Zurich Switzerland; 4 Department of Psychiatry, Psychotherapy and Psychosomatics University of Zurich Zurich Switzerland; 5 Center for Technology and Behavioral Health Departments of Biomedical Data Science and Psychiatry Geisel School of Medicine, Dartmouth College Hanover, NH United States; 6 Future Health Technologies Singapore-ETH Centre Campus for Research Excellence And Technological Enterprise Singapore Singapore; 7 Lee Kong Chian School of Medicine Nanyang Technological University Singapore Singapore; 8 Department of Primary Care and Public Health School of Public Health Imperial College London London United Kingdom

**Keywords:** depression, digital mental health, smartphone applications, just-in-time adaptive interventions, effectiveness, mobile phone

## Abstract

**Background:**

The number of smartphone apps that focus on the prevention, diagnosis, and treatment of depression is increasing. A promising approach to increase the effectiveness of the apps while reducing the individual’s burden is the use of just-in-time adaptive intervention (JITAI) mechanisms. JITAIs are designed to improve the effectiveness of the intervention and reduce the burden on the person using the intervention by providing the right type of support at the right time. The right type of support and the right time are determined by measuring the state of vulnerability and the state of receptivity, respectively.

**Objective:**

The aim of this study is to systematically assess the use of JITAI mechanisms in popular apps for individuals with depression.

**Methods:**

We systematically searched for apps addressing depression in the Apple App Store and Google Play Store, as well as in curated lists from the Anxiety and Depression Association of America, the United Kingdom National Health Service, and the American Psychological Association in August 2020. The relevant apps were ranked according to the number of reviews (Apple App Store) or downloads (Google Play Store). For each app, 2 authors separately reviewed all publications concerning the app found within scientific databases (PubMed, Cochrane Register of Controlled Trials, PsycINFO, Google Scholar, IEEE Xplore, Web of Science, ACM Portal, and Science Direct), publications cited on the app’s website, information on the app’s website, and the app itself. All types of measurements (eg, open questions, closed questions, and device analytics) found in the apps were recorded and reviewed.

**Results:**

None of the 28 reviewed apps used JITAI mechanisms to tailor content to situations, states, or individuals. Of the 28 apps, 3 (11%) did not use any measurements, 20 (71%) exclusively used self-reports that were insufficient to leverage the full potential of the JITAIs, and the 5 (18%) apps using self-reports and passive measurements used them as progress or task indicators only. Although 34% (23/68) of the reviewed publications investigated the effectiveness of the apps and 21% (14/68) investigated their efficacy, no publication mentioned or evaluated JITAI mechanisms.

**Conclusions:**

Promising JITAI mechanisms have not yet been translated into mainstream depression apps. Although the wide range of passive measurements available from smartphones were rarely used, self-reported outcomes were used by 71% (20/28) of the apps. However, in both cases, the measured outcomes were not used to tailor content and timing along a state of vulnerability or receptivity. Owing to this lack of tailoring to individual, state, or situation, we argue that the apps cannot be considered JITAIs. The lack of publications investigating whether JITAI mechanisms lead to an increase in the effectiveness or efficacy of the apps highlights the need for further research, especially in real-world apps.

## Introduction

### Background

Globally, each year, at least 246 million people are affected by depression [1], and depression is the leading cause of years lived with disability [2]. Although effective treatments for depression exist [3-5], most individuals in need still do not receive effective treatment [6], or those obtaining treatment do not benefit. People seeking help often face barriers such as the high costs of treatment, a shortage of trained clinicians, the stigma associated with seeking help, and accessibility difficulties [7-10].

Mobile apps may have the potential to address the rising prevalence of depression and the insufficient resources available for treatment [11,12]. Apps are already an integral part of most people’s everyday lives [13], and the threshold for engagement with apps is assumed to be low, resulting in prompt, flexible, portable, and anonymous treatment for people with depression [14]. Individuals otherwise not reachable could receive treatment [15], and the interventions could be delivered in economies that have limited resources for mental health treatment [16]. Small to large effect sizes have been reported by several systematic reviews that show that apps and other digital interventions reduce symptoms of mental health problems, including depression [17-20]. Finally, apps can be used in real-life situations, where behavior change is most desirable and clinicians are unable to intervene [14].

### Objective

This study aims to complement the existing assessment of apps that address depression by focusing on the use of just-in-time adaptive intervention (JITAI) mechanisms [21,22]. JITAIs aim to deliver an adaptive treatment (ie, personalized or tailored) at a time of vulnerability (ie, “person’s transient tendency to experience adverse health outcomes or to engage in maladaptive behaviors” [21]) and receptivity (ie, “the person’s transient tendency to receive, process, and use the support provided” [21]). To be considered a JITAI, an intervention needs to deliver the content when a state of vulnerability and state of receptivity are detected. Vulnerability refers to *the right time,* that is, a moment of heightened exposure or receptiveness for a negative health outcome [23]. For example, a JITAI that addresses depression should deliver an intervention component when changes in mood are detected. Ideally, the component delivered should aim to improve the symptom that has been detected. The treatment must not only be delivered at *the right time* (*vulnerability)* but the recipient also needs to be *receptive* (*receptivity)*. Receptivity refers to *the availability*, that is, the detection of the time when an individual can receive, process, and use the support provided [23]. For a JITAI that addresses depression, this could be the time when the individual is at home because delivery of a behavior activation component needs a safe space.

The measured outcomes used to tailor content and timing along a state of vulnerability and state of receptivity are referred to as tailoring variables [21,22]. Although ecological momentary assessments may facilitate the detection of these tailoring variables, passive measurements (eg, using the location derived from a smartphone’s GPS data) are regarded as the gold standard of tailoring variables for JITAIs. These passive measurements have the advantage of enabling unobtrusive, continuous observation [23]. Although tailoring content and timing to the state of receptivity and state of vulnerability may be possible by asking ecological momentary assessments (eg, asking through message, “Would now be a good time to start an activity?”), the benefit of passive measurements may be that the content and timing are tailored without deliberate interaction with the intervention itself (eg, detecting a state of vulnerability and state of receptivity and sending a notification, “It seems that you are at home and in a bad mood. Would you like to start an exercise?”). Therefore, it has been proposed that JITAIs that tailor the content to the person, situation, and time by using these passive measurements reduce the burden on users and increase the effectiveness of the intervention [21,22].

Evidence of the higher effectiveness of JITAIs compared with that of non-JITAI treatments and wait-list control groups was investigated in a recent meta-analysis [24], which found moderate to large effect sizes (Hedges *g*=1.65 when compared with wait-list control and Hedges *g*=0.89 when compared with non-JITAI treatments) of the primary outcomes produced by 33 empirical studies. Owing to the potential of JITAIs to increase effectiveness while reducing the burden on users and the prominence of the JITAI framework in the scientific community, the aim of this study is to determine the degree to which popular apps that address depression use JITAI mechanisms by reviewing which relevant symptoms of depression (eg, mood) are measured as well as how they are measured. We are also interested in learning whether peer-reviewed studies can be found that investigated the increased effectiveness or efficacy of these apps attributable to the use of JITAI mechanisms.

To this end, we systematically assessed popular apps that target depression, that is, apps that have the most reviews on the Apple App Store and most downloads on the Google Play Store. We argue that the investigation of JITAI mechanisms is necessary because of their potential to increase effectiveness while simultaneously decreasing the burden on users. The focus on popular apps is important because they are listed at the top of search results and thus are very likely to be downloaded and used [25]. Moreover, a high number of downloads implies that they have been found to be useful by users [26] and may indicate that people continue to use or recommend them. Recent evidence also indicates that the 2 most popular apps for depression and anxiety were responsible for 90% of the 13.3 million active monthly users (Headspace with 6,959,000 users equivalent to 52% of the active users and Calm with 5,000,000 users equivalent to 38% of the active users) [27]. Although the review by Wasil and Gillespie [27] focused on depression and anxiety apps, similar user statistics should be observed when focusing only on apps for depression.

## Methods

### Search Strategy and Selection Criteria

We conducted this systematic app search and literature review following the methods used in existing reviews [26,28-30] of popular apps that address mental health problems. We systematically identified and reviewed apps that were publicly available in the US and UK app stores because number of venture capital backed digital health deals in the first half of 2020 in these countries ranked first (United States: 475) and second (United Kingdom: 41) of all English-speaking countries [31]. The Apple App Store and Google Play Store were used because they have a combined market share of approximately 99.4% [32]. We searched the two stores by entering the term *depression* in the search fields of the stores and included all apps found in both stores of both countries. We also reviewed curated lists of health apps from prominent organizations, namely the Anxiety and Depression Association of America [33], the National Health Service [34], and the American Psychological Association [35], to ensure that we did not miss any app recommended by important institutions and experts for mental health. The apps found in these lists addressed several different mental health problems. We selected only those apps that addressed depression for further assessment. The searches were carried out in August 2020. All apps found regardless of the category in which they were found (eg, lifestyle), download category, or number of reviews were included in the initial list of apps. This list of all the apps found was screened according to the inclusion and exclusion criteria outlined below.

For further assessment, we included the apps that targeted the treatment of depression or reduction of the symptoms of depression by delivering at least one active ingredient and were available in English. We defined an active ingredient, on the lines of the study by Michie et al [36], as a function supporting users in their management of depression that is designed to reliably and causally change processes that govern behavior [37]. An example of an active ingredient for depression could be a goal-setting task, a breathing exercise, or a recording of daily mood. Apps targeting other mental health illnesses such as anxiety or posttraumatic stress disorder were not excluded if depression was addressed as well. We included both free and paid apps. Browser-based treatments were not included. We excluded apps that only targeted professionals (eg, Depression Psychopharmacology), only offered a diagnostic service (eg, Patient Health Questionnaire-9 Depression Test Questionnaire), only provided quotes or inspirational text (eg, Depression Quote Wallpapers), or only conveyed information without the goal of eliciting behavior change or engaging with individuals (eg, Psychology Book: 1000+ Amazing Psychology Facts).

Keeping the inclusion and exclusion criteria in mind, 2 authors (GWT and ADF) separately reviewed each app identified from the initial search for depression apps in the two stores and the curated lists. The interrater agreement was excellent (Cohen κ=0.91). In case of disagreements, a consensus was reached through discussion. After this initial assessment, we ranked all included apps from the Apple App Store separately by their number of reviews and all included apps from the Google Play Store by their download category (eg, 1,000,000+ and 500,000+ downloads). We decided to screen the apps before ranking them to ensure that the most popular apps for depression—and not the most popular apps from our initial lists—were included in the analysis.

Next, we separately identified the most popular apps available only in the Apple App Store, available only in the Google Play Store, or available in both stores. For apps only available in the Apple App Store, we selected the 5 most reviewed apps because users rarely scroll past the first 5 apps on the list [25]. For the Google Play Store, we used the download category of the app ranked fifth on the list (eg, 500,000+ downloads). All apps in the 500,000+ download category were included. For apps available in both app stores, we used the Google Play Store’s 500,000+ download category to determine inclusion, regardless of the number of reviews on the Apple App Store. Regardless of the number of downloads or reviews, we included all apps from the curated lists that met the inclusion criteria and did not violate the exclusion criteria.

### Data Analysis

Our evaluation covered the following areas: general information about the app, potential mechanisms for delivery of the JITAI, and peer-reviewed evidence. We developed our evaluation framework before reviewing the apps and used Covidence Systematic Review software (Veritas Health Innovation Ltd; version accessed in August 2020) to review the apps. All the questions are listed in the codebook in Tables S1-S6 of Multimedia Appendix 1 [21,23,38-48], along with the sources from which we derived them. Each included app was evaluated separately by 2 raters (GWT and ADF) as follows:

First, we gathered general information about the apps, including the name of the provider, additional affiliated organizations (eg, other companies, universities, governments, or nongovernmental organizations), and time since last update. Second, we reviewed the app’s website and recorded all the publications provided as well as information about JITAIs. Third, we searched for peer-reviewed publications in PubMed, Cochrane Register of Controlled Trials, PsycINFO, Google Scholar, IEEE Xplore, Web of Science, ACM Portal, and Science Direct using the search term *[(Name AND App) OR (Name AND Application AND Smartphone)]*.

Fourth, we reviewed the full text of each study found on the website and in the different databases. We excluded books, theses, systematic reviews evaluating several different apps, and clinical trial registrations. Subsequently, each study was evaluated in line with prior work [38], including the year of publication, journal name, journal impact factor, the number of subjects, study purpose, and study design (ie, randomized controlled trial or open trial). We also extracted the information available about *the JITAI mechanism*. We determined the degree to which the apps could be considered *JITAIs* by reviewing whether, and to what degree, the relevant features derived from the JITAI concept described in the study by Nahum-Shani et al [22] had been implemented. In the first step, we reviewed how potential tailoring variables were measured. In the second step, we reviewed whether these measurements were used to determine a state of vulnerability (ie, a moment of heightened exposure or receptiveness for a negative health outcome) or state of receptivity (ie, the time when the input could be perceived, processed, and used). To leverage the potential of JITAIs, the state of vulnerability and state of receptivity not only need to be detected through measurements but the treatment content and timing also need to be tailored along these measurements. We assessed how the tailoring variables were measured by reviewing the *symptoms of depression* that were measured (derived from the *International Classification of Diseases, 10th Revision* and *Diagnostic and Statistical Manual of Mental Disorders, Edition 5*) and the *self-report* data or *sensor and device analytics* (derived from related work [23] from the Android Developers Guide [39] and the iPhone operating system Security Guide [40]) that were used. We also reviewed whether tailoring to traits (ie, “tailoring-to-people” [21]) was used by checking for questions about demographics and socioeconomic status. Furthermore, we checked whether receptivity was measured by GPS location, self-reports, or by measuring symptoms. As it has been proposed that JITAI mechanisms increase the effectiveness or efficacy of such apps [22], we reviewed whether the publications addressed effectiveness or efficacy and whether JITAI mechanisms were investigated in these publications.

**Figure 1:**
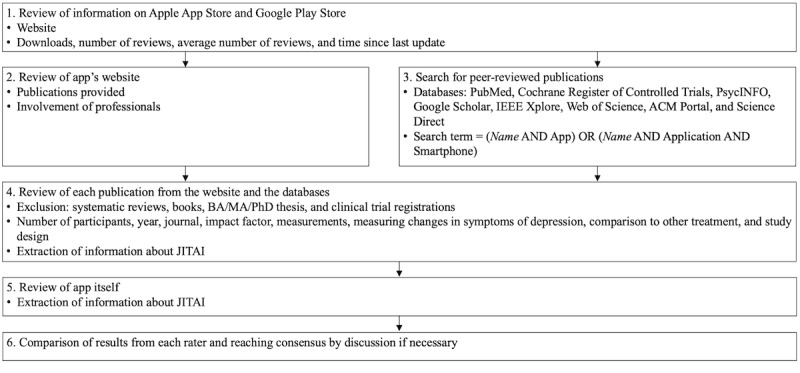
Review process for each app. BA: bachelor; JITAI: just-in-time adaptive intervention; MA: master; PhD: doctor of philosophy.

Finally, we reviewed the app itself and extracted the information available about JITAI mechanisms. The results from each rater were compared, and consensus was reached by discussion, if necessary. We reviewed each app in September 2020, and the process is illustrated in Figure 1.

## Results

### Apps

**Figure 2:**
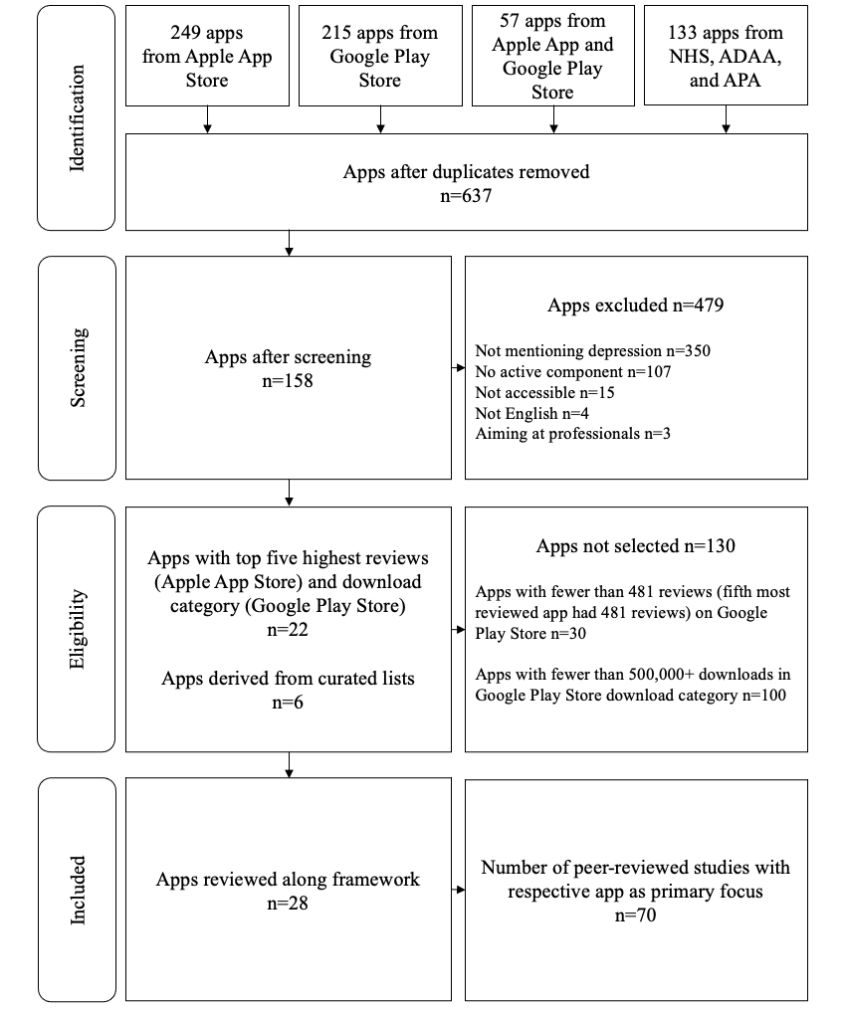
Inclusion and exclusion process of reviewed apps. ADAA: Anxiety and Depression Association of America; APA: American Psychological Association; NHS: National Health Service.

We found 249 apps in the Apple App Store, 217 apps in the Google Play Store, 57 apps in both stores, and 135 apps in the curated list, yielding a total of 658 apps. Of the 658 apps, we removed 17 (2.6%) duplicates, 349 (53%) apps that did not mention depression, 123 (18.7%) apps with no active component, 8 (1.2%) apps that were not accessible, 1 (0.2%) app not available in English, and 1 (0.2%) app that targeted professionals. The reasons for excluding each app are presented in Table S7 of Multimedia Appendix 1. The reasons for excluding apps because of missing active ingredients were also summarized and are presented in Table S8 of Multimedia Appendix 1 with further details. We ranked the apps found only in the Apple App Store based on their number of reviews and included the 5 most reviewed apps. We ranked the remaining apps found in the Google Play Store and in both stores according to their number of downloads category. The fifth most downloaded app in the Google Play Store fell in the 500,000+ download category. Therefore, we included all apps found in the Google Play Store and all apps available in both Apple App Store and Google Play Store with more than 500,000+ downloads, yielding 17 apps. We included 6 apps from the curated lists that met the inclusion criteria of mentioning depression and did not violate the exclusion criteria, yielding a total of 28 apps. A flowchart of the results of the review process is presented in Figure 2.

Using methods similar to those used in the study by Baumel et al [49], we calculated the number of users and reviews covered by our selection of the top 5 reviewed apps from the Apple App Store and apps with more than 500,000 downloads found in the Google Play Store and apps found in both the Apple App Store and Google Play Store. The calculations are presented in Tables S9 and S10 of Multimedia Appendix 1 (Table S9 for apps ranked by their download category and Table S10 for apps ranked according to their number of reviews). Using the cutoff point of the 5 most reviewed apps, we included 91% of the reviews of the 35 apps found only in the Apple App Store that met the inclusion criteria. Including the sixth most reviewed app would have resulted in an increase of 1.97% of the reviews in the sample. By including apps with more than 500,000 downloads (ie, the 500,000+ download category), we included 96.7% of the users of the 118 apps found in the Google Play Store and both Google Play Store and Apple App Store that remained after screening. Including the download category of more than 100,000 downloads would have resulted in an increase of 3% in the downloads in the sample.

### Publications

We found 70 peer-reviewed publications for the 28 reviewed apps (Multimedia Appendix 1 TableS12) [50-119]. We found at least one publication for 57% (16/28) of the apps, at least one peer-reviewed publication investigating the effectiveness of 32% (9/28) of the apps, and at least one peer-reviewed publication investigating the efficacy of 18% (5/28) of the apps. Of the 70 publications, although 23 (33%) investigated the effectiveness of the apps, and 14 (20%) investigated their efficacy, no publication evaluated an increase in effectiveness or efficacy achieved by using JITAI mechanisms. The extracted information from all the reviewed publications is presented in Tables S11 and S12 of Multimedia Appendix 1.

### Included Apps

The 28 apps included were rated 2,808,465 times, with each app being rated on average 100,302.32 times (SD 279,723.5; range 127-1,229,623; median 8,109, IQR 28,310). The average rating for all apps was 4.44 stars (SD 0.34; range 3.40-4.95; median 4.50, IQR 0.37) out of a possible five stars. The number of reviews and average reviews were calculated using weighted means with information from the Apple App Store and Google Play Store, if available. Table 1 lists the general information and JITAI mechanisms of each reviewed app and Table 2 summarizes the 28 apps that were included in the review and evaluated.

**Table 1:** General information and JITAI^a^ mechanisms of the reviewed apps (N=28).

App	Operating system	Number of reviews	AVR^b^	NODC^c^	Symptoms measured and measurements used	NDS^d^ used	NSR^e^ used	NSDA^f^ used
Calm	AND^g^, iOS^h^	1,229,623	4.71	10,000,000+	Activity, mood, unhelpful beliefs, sleep, open questions, multiple-choice questions	4	2	—^i^
Headspace	AND, iOS	872,025	4.86	10,000,000+	—	—	—	—
Daylio	AND, iOS	328,048	4.62	10,000,000+	Activity, mood, appetite, sleep, ratings, multiple-choice questions	4	2	—
Youper	AND, iOS	61,478	4.67	1,000,000+	Activity, mood, interest or pleasure, sleep, vital signs, location, open questions, ratings, multiple-choice questions	4	3	2
Moodpath	AND, iOS	57,011	4.64	1,000,000+	Activity, mood, interest or pleasure, appetite, unhelpful beliefs, concentration, sleep, vital signs, open questions, ratings, multiple-choice questions	7	3	1
Wysa	AND, iOS	56,520	4.70	1,000,000+	Activity, mood, interest or pleasure, unhelpful beliefs, concentration, ratings, multiple-choice questions	5	2	—
Friend Shoulder	AND	32,847	4.30	1,000,000+	Mood, unhelpful beliefs, suicidal thoughts, open questions	3	1	—
BetterHelp	AND, iOS	30,592	4.63	500,000+	Mood, unhelpful beliefs, suicidal thoughts, open questions	3	1	—
Sanvello	AND, iOS	27,536	4.67	1,000,000+	Activity, mood, interest or pleasure, unhelpful beliefs, suicidal thoughts, sleep, microphone, open questions, ratings	6	2	1
7 Cups	AND, iOS	20,709	4.29	1,000,000+	Mood, interest or pleasure, cognition, unhelpful beliefs, distorted perception, open questions, ratings	5	2	—
Control and Monitor	AND	16,149	4.50	1,000,000+	Activity, mood, appetite, unhelpful beliefs, suicidal thoughts, sleep, ratings, multiple-choice questions	6	2	—
#SelfCare	AND, iOS	14,670	4.57	500,000+	—	—	—	—
Remente	AND, iOS	11,494	4.34	1,000,000+	Activity, mood, open questions, ratings, multiple-choice questions	2	3	—
Reflexio	AND	8118	4.30	1,000,000+	Mood, open questions, ratings	1	2	—
Moodnotes	iOS	8100	4.70	—	Mood, illogical thinking, camera, open questions, ratings, multiple-choice questions	2	3	1
Online Therapy—Mental Help	AND	5979	4.40	500,000+	Mood, unhelpful beliefs, suicidal thoughts, open questions	3	1	—
InnerHour	AND, iOS	5402	4.50	500,000+	Activity, mood, interest or pleasure, unhelpful beliefs, concentration, suicidal thoughts, sleep, ratings, multiple-choice questions	8	2	—
Happify	AND, iOS	5164	4.21	500,000+	Activity, mood, interest or pleasure, cognition, unhelpful beliefs, sleep, illogical thinking, distorted perception, vital signs, camera, open questions, ratings, multiple-choice questions	8	3	2
What's Up?—A Mental Health App	AND, iOS	3446	4.22	500,000+	Activity, mood, interest or pleasure, open questions, ratings, multiple-choice questions	3	3	—
MoodTools—Depression Aid	AND, iOS	3167	4.31	100,000+	Activity, mood, interest or pleasure, appetite, cognition, unhelpful beliefs, concentration, suicidal thoughts, sleep, distorted perception, ratings, multiple-choice questions, Patient Health Questionnaire	10	3	—
DBT^j^ Coach	AND, iOS	3067	4.95	10,000+	Activity, mood, interest or pleasure, appetite, unhelpful beliefs, concentration, suicidal thoughts, sleep, ratings, multiple-choice questions, Patient Health Questionnaire	8	3	—
CBT^k^ Thought Diary	AND, iOS	2182	4.58	100,000+	Mood, distorted perception, open questions, ratings, multiple-choice questions	2	3	—
T2 Mood Tracker	AND, iOS	1873	3.40	100,000+	Activity, mood, cognition, unhelpful beliefs, concentration, sleep, open questions, ratings	6	2	—
Joyable	AND, iOS	1522	4.48	5000+	Activity, mood, interest or pleasure, appetite, unhelpful beliefs, suicidal thoughts, sleep, open questions, ratings, multiple-choice questions	7	3	—
Breeze	iOS	1313	4.70	—	Activity, mood, ratings, multiple-choice questions	2	2	—
Moodkit	iOS	159	4.40	—	Activity, mood, appetite, sleep, open questions, ratings, multiple-choice questions	4	3	—
Catch It	AND, iOS	144	3.66	50,000+	Mood, open questions, ratings, multiple-choice questions	1	3	—
Feeling Good	AND, iOS	127	3.91	10,000+	—	—	—	—

^a^JITAI: just-in-time adaptive intervention.

^b^AVR: average rating out of five possible stars.

^c^NODC: number of downloads category.

^d^NDS: number of depression symptoms.

^e^NSR: number of self-reports.

^f^NSDA: number of sensors and device analytics.

^g^AND: Android operating system.

^h^iOS: Apple operating system.

^i^App did not measure a symptom, did not use a self-report, or did not use sensors and device analytics.

^j^DBT: dialectical behavior therapy.

^k^CBT: cognitive behavioral therapy.

**Table 2:** Summary of the number of reviews, average rating, download category, and JITAI^a^ mechanisms used by the reviewed apps (N=28).

	Sum	Count	Value, mean (SD; range)	Value, median (IQR)
Reviews	2,808,465	28	100,302.32 (279,723.50; 127-1,229,623)	8109 (28,310)
Rating^b^	—^c^	28	4.44 (0.34; 3.40-4.95)	4.50 (0.37)
Download category	42,375,000	25	1,695,000 (3,153,748.43; 5000-10,000,000)	500,000 (900,000)
Depression symptoms	114	25	4.56 (2.47; 1-10)	4 (3)
Self-reports	59	25	2.36 (0.7; 1-3)	2 (1)
Sensors and device analytics	7	5	1.4 (0.55; 1-2)	1 (1)

^a^JITAI: just-in-time adaptive intervention.

^b^Out of five possible stars.

^c^Not available.

### JITAI Mechanisms

**Figure 3:**
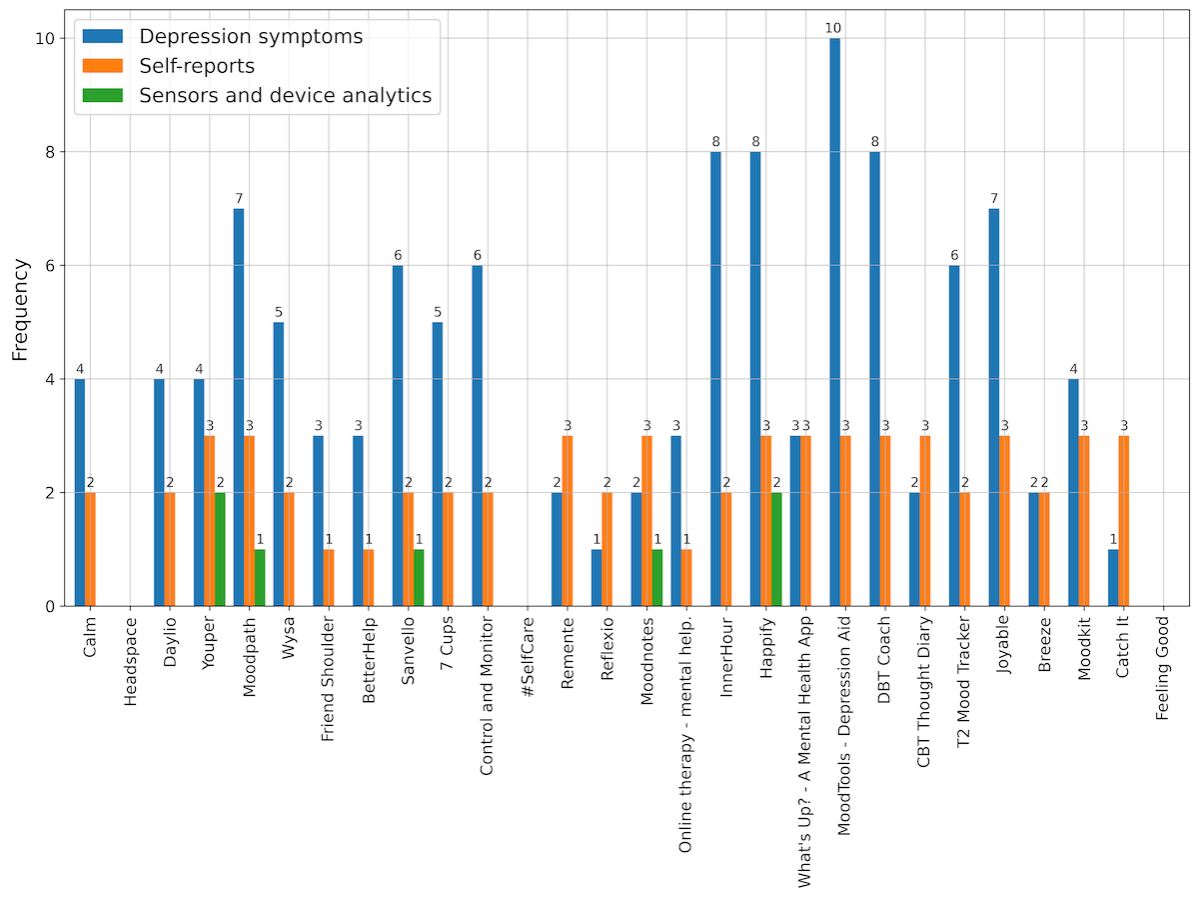
Depressive symptoms measured and frequency of measurements used for each of the 28 reviewed apps. CBT: cognitive behavioral therapy; DBT: dialectical behavior therapy.

We found that, of the 28 reviewed apps, 25 (89%) measured some kind of *depression symptom* when users interacted with the app (eg, initial assessment when starting the app), whereas 3 (11%) did not use any measurements, 20 (71%) used at least one s*elf-report* (eg, daily report of mood changes through a rating), and 5 (18%) used *self-reports* and *sensors and device analytics* (eg, tracking activity through location data). Figure 3 illustrates the number of *depression symptoms* measured by different *self-reports* or *sensors and device analytics* for each of the reviewed apps. *Mood Tools*—*Depression Aid* measured the most *depression symptoms* (10 different symptoms measured) while not using any *sensors and device analytics*. *Happify* and *Youper* measured fewer *depression symptoms* (eight and four, respectively) but used two different *sensors and device analytics*.

**Figure 4:**
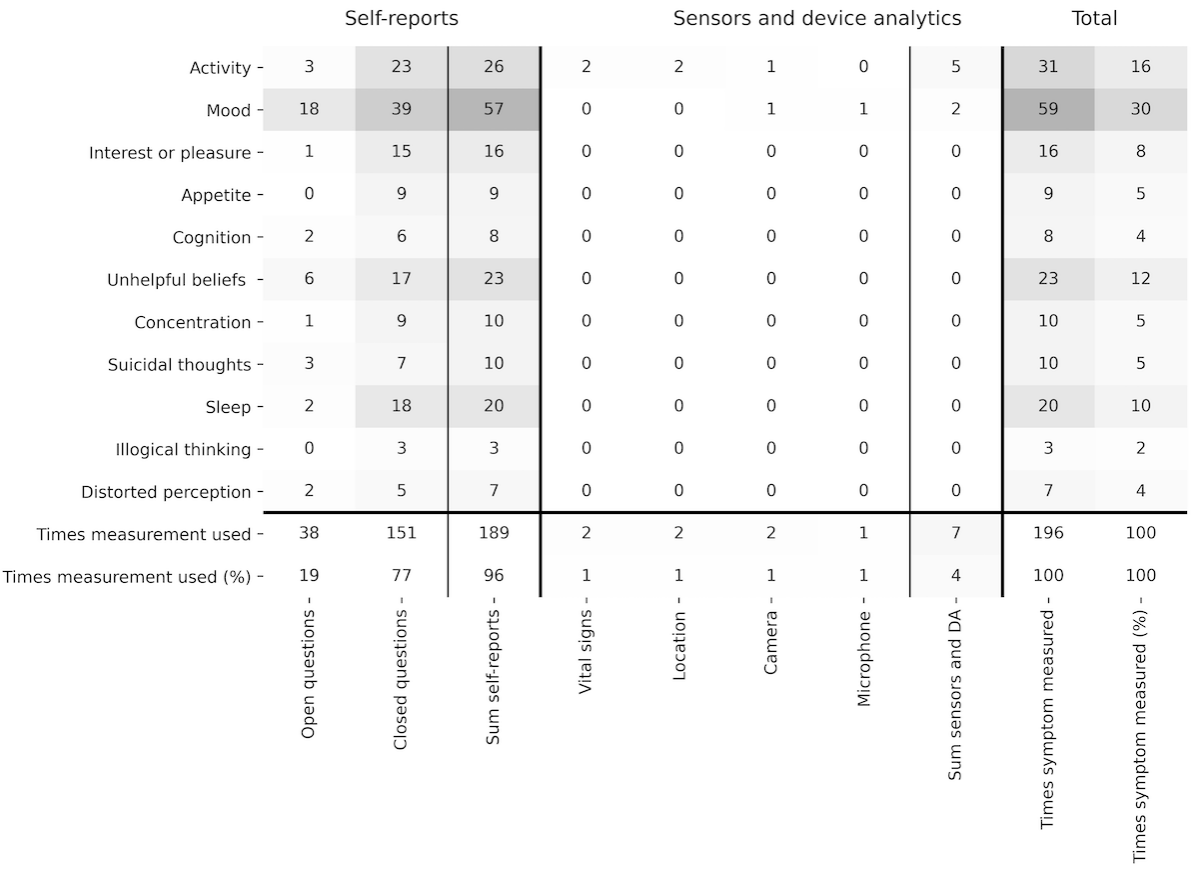
Heatmap of measurements used to measure symptoms. The heatmap illustrates the number of times that symptoms of depression were measured by self-reports or sensors and device analytics, summarized over the 28 reviewed apps. A darker color illustrates a higher number of occurrences, also indicated by the annotation in the cells. DA: device analytics.

Our findings regarding the use of *self-reports* and *sensors and device analytics* are summarized in Figure 4. We found that a symptom was measured by a *self-report* or *sensors and device analytics* 196 times. For example, the app *Calm* measured four symptoms (activity, mood, unhelpful beliefs, and sleep) by using two different self-reports for each symptom (open questions and multiple-choice questions), resulting in a symptom measurement rate of 4.1% (8/196). To measure different *depressive symptoms*, *self-reports* were used almost exclusively (189/196, 96.4%), and *sensors and device analytics* were used rarely (7/196, 3.6%). The s*elf-reports* used most frequently to measure different *depressive symptoms* were *closed questions* consisting of ratings (eg, rate your mood from 1 to 10), Likert scales (eg, indicate how often you felt sad last week, with the following options: never, almost never, sometimes, most of the time, and always), and multiple-choice questions (eg, select the activities that made you happy in the last week) for a measurement rate of 77% (151/196). The measurement rate for *open questions* (ie, questions without a fixed response or text field with open input) was 19.4% (38/196). Of the 3.6% (7/196) using *sensors and device analytics*, the most frequently used were *vital signs* (mostly heart rate), *location* and camera with each 0.5% (1/196) measurement rate. The measurement rate for the symptom *mood* (most frequently measured symptom) was 30.1% (59/196), for *activity* 15.8% (31/196), for *unhelpful beliefs* 11.7% (23/196), and for s*leep* 10.2% (20/196).

**Figure 5:**
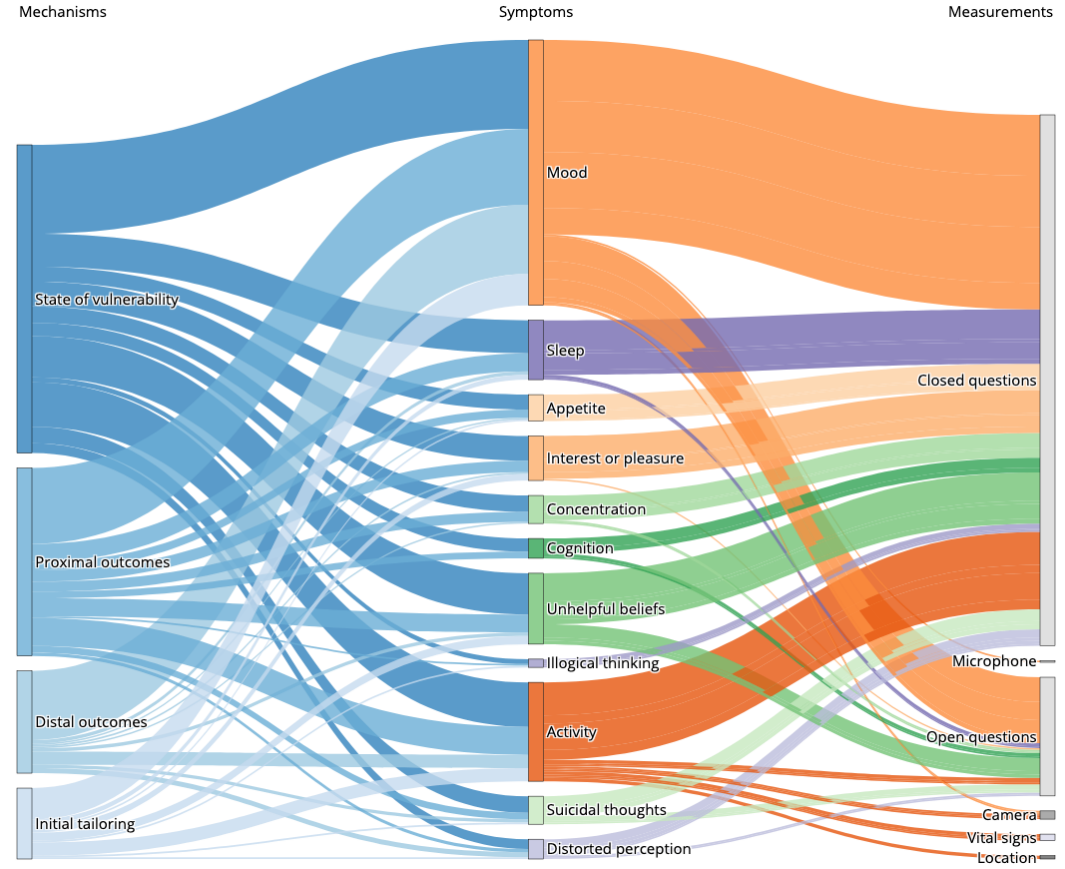
Connection among just-in-time adaptive intervention (JITAI) mechanisms, symptoms, and measurements. Sankey diagram illustrating the different JITAI mechanisms (state of vulnerability, proximal outcomes, distal outcomes, and tailoring variables) for which we were able to match a depressive symptom (eg, mood) and the measurements used to capture the changes (eg, closed question). The JITAI mechanisms are displayed in blue; depressive symptoms in orange, green, and purple; and measurements in gray. The size of the rectangle indicates the number of times that the mechanism, symptom, or measurement was found. The thickness of the connection indicates the number of times that a measurement or symptom was used. Some measurements have been assigned to two or three JITAI mechanisms; therefore, double counting is possible.

When possible, we tried to match the measurement of the depressive symptom to a mechanism relevant to the JITAI concept. We were able to do so for s*tate of vulnerabilit*y, *proximal outcomes*, *distal outcomes*, and *initial tailoring*. Some of the measurements could have been used as tailoring variables for two or three *JITAI* mechanisms. Therefore, it is possible that double counting of the symptoms and measurements for each mechanism occurred. Figure 5 illustrates our findings, including which measurements were used to measure which symptom and for which *JITAI* feature. The figure shows that some s*ensors and device analytics* were not used as passive measurements; rather, they were used to actively capture changes. For example, the *camera* was used as a measurement for *activity,* with users being asked to take pictures of locations that they had visited or a picture of something that made them sad to describe their *mood*.

## Discussion

### Principal Findings

We reviewed the 28 most popular or recommended apps for depression found in the Apple App Store, Google Play Store, and in curated lists compiled by respected mental health authorities. Regarding our main aim to investigate JITAI mechanisms, we found that none of the reviewed apps specifically mentioned the use of JITAI mechanisms in the app, on their websites, or in the identified peer-reviewed publications. Of the 28 apps, we found that 3 (11%) did not use any measurements, and 20 (71%) only used *self-reports* (189/196, 96.4% of all measurements were self-reported). Although such *self-reports* can be used as *in-the-moment assessments* (ie, ecological momentary assessments) that are closely related to the JITAI concept [24], we argue that they are insufficient for leveraging the full potential of JITAIs. Of the 28 apps, we found that 5 apps (18%) also used *sensors and* *device analytics* (7/196, 3.6% of all measurements involved *sensor* *and* *device analytics*). However, we found that most *sensors* such as the *camera* were used as *in-the-moment* assessments or as part of an app’s features; they were not used to tailor the content or timing. Some of the apps measured *depressive symptoms* by using s*elf-reports* when the app was first opened to determine what kind of content should be presented (eg, measuring the need to focus on sleep or mood). However, these measurements were not used to tailor the content that was presented afterward. Furthermore, static tailoring, which has been observed to be less effective than dynamic tailoring [120], is, in our view, not sufficient for an app to be considered as a JITAI. Relying on this initial assessment may also be insufficient because symptoms of depression seem to change frequently through the day and may be highly specific to an individual [121].

Interestingly, we found that besides *mood* (symptom measurement rate of 59/196, 30.1%) and *decreased activity* (symptom measurement rate of 31/196, 15.8%), other symptoms of depression were measured less frequently. Given the broad variety and severity of depression [122] and the high comorbidity with other mental health problems such as anxiety [123], this focus on a subset of symptoms may not be sufficient to detect changes that might indicate a need for support. In addition, a focus on the improvement of the main symptoms (eg, *mood* and *activity,* derived from the *Diagnostic and Statistical Manual of Mental Disorders, Edition 5*) may not be sufficient to contribute to the understanding of the complex processes involved in depression. Accurate and continuous measurements of psychophysiological changes enabled by passive measurements of various physiological features (eg, changes in breathing patterns or vital signs) may, however, improve the understanding of depression in general. Such an understanding could, in turn, enable an even more successful implementation of JITAIs.

Related work has shown that user adherence to various mental health apps dropped substantially after interacting with the app for only a couple of days [49]. Although details of user engagement with the apps reviewed in the study by Baumel et al [49] were not available, several apps that were included in our study were also included in this review (eg, *Headspace*, *Calm*, *Daylio*, *7Cups*, *Happify*, *Moodpath*, and *T2 Mood Tracker*). These reviewed apps showed a drastic decline in user numbers after a couple of days, highlighting the importance of finding ways to increase user adherence. A proposed key advantage of a JITAI is to address this challenge of declining adherence by offering support in times of need and reducing the burden on the individual. An app that solely relies on self-reports entered by the user while interacting with the app cannot achieve this goal. Individuals would need to open the app and engage with it to enter the responses before tailoring to the right time could be carried out. Continuously requiring such self-reports may even be counterproductive to adherence because of the increase in the burden. Therefore, future work should address the extent to which JITAIs that tailor content along passive measurements are capable of increasing adherence. A comparison among apps that do not use any measurements, that use static tailoring to measurements, that use tailoring to self-reports only, or that use tailoring to both self-reports and passive measurements would be necessary to determine the benefit of dynamic tailoring with regard to increasing user adherence.

Considering self-reported questions further by focusing on open questions, the question arises as to what degree the answers to these questions could be used to tailor content without any further use of analytics. Although self-reported closed questions already suffer from some biases (eg, recall bias and biases due to mental illness), this shortcoming is addressed by using different questions to infer an underlying construct and to validate questionnaires in different samples. In contrast, open questions may still be limited to measuring symptoms of depression and need to rely on methods derived from natural language processing to cope consistently and accurately with the large amount of data entered. Contrary to closed-question questionnaires, these methods have not been validated as rigorously as existing questionnaires for symptoms and symptom severity. Therefore, it is unclear to what degree open questions can be used to measure symptoms of depression. Existing work using free text has focused on using data from other sources to measure symptom changes. In a recent review by Chancellor and De Choudhury [124], 75 such studies were investigated. In these studies, social media data were used to infer various mental health problems. The authors share our concern regarding consistency and accuracy by concluding that construct validity and lack of reflection may limit the ability to use text to measure health status. In contrast, passive measurements that could be derived when users answer open questions, such as keystroke frequency or pressure on display, have been used to measure cognitive functions associated with mental health [125].

As stated in the Introduction section, the JITAI concept aims to tailor content and timing based on the measured state of vulnerability and state of receptivity. Our results indicate that the measurements used in the reviewed apps may only serve for tailoring to the state of vulnerability (eg, mood and activity). We did not find any measurement that may serve to tailor to the state of receptivity. Plausible examples of such receptivity tailoring are still conceivable for both self-reports (eg, asking whether an individual is alert enough to read a text or complete a demanding exercise) and passive measurements (eg, detecting the user’s arrival at home through GPS data and time of day to trigger a journaling exercise). In general, studies investigating possible markers to detect receptivity and the effectiveness of such receptivity tailoring are sparse. This research gap becomes even more evident when one considers the various studies that aim to develop passive measurements that could be used to determine vulnerability [121,126-132] together with the finding that *tailoring to what* had a significant impact on the effectiveness of JITAIs [24].

Other concepts related to receptivity, such as interruptibility [133], have been investigated in greater detail [134]. In accordance with the study by Künzler et al [135], we would still argue that receptivity extends these concepts by combining concepts such as the willingness to receive an intervention (interruptibility), engage with the intervention, and process the provided information. To the best of our knowledge, only the following studies have specifically focused on investigating receptivity. The study by Künzler et al [135] developed machine learning models to predict receptivity in a physical activity intervention. In the study by Choi et al [136], the authors describe the results of a 3-week study that they conducted to investigate the context and cognitive or physical state to understand the response to the components of sedentary behavior. The study by Sarker et al [137] used the response to ecological momentary assessments—but not to an intervention component—to explore discriminative features for building machine learning models to detect receptivity and reported 77.9% accuracy. Although these studies used post hoc analysis to determine receptivity, the study by Mishra et al [134] used data from the study by Künzler et al [135] to test the two different models in a real-world study. The authors found that receptivity increased by 40% compared with the control when a static machine learning model was used and that a dynamic machine learning model led to an increase in receptivity over time, whereas receptivity in the control conditions declined. These results are promising because they show that with relatively easily derived information such as time of day or Wi-Fi connection, receptivity and engagement can be maintained, or engagement can even be increased over time, which is something that mental health interventions have been observed to struggle with [49].

The finding that most measurements could have been used to tailor to the state of vulnerability but not to the state of receptivity may be explained by three considerations. First, as shown in the study by Wang and Miller [24], the *tailoring to what* (ie, both previous behavioral patterns of people and their current need states) showed significant improvement. However, results regarding the *tailoring to when* have not been reported. Second, most related work aimed at developing markers for detecting changes in symptom severity or depressive severity [121,126-132]. Although some of these outcomes may also be relevant for the state of receptivity, the primary use of these measurements would be to determine the state of vulnerability (eg, mood changes detected by voice), distal outcome (eg, depression severity score calculated by combining different symptom outcomes), and proximal outcome (eg, sleep quality measured through vital signs). Finally, as mentioned above, it seems that receptivity as a concept has been investigated less often, with related work focusing on concepts such as interruptibility [133] or detection of boredom [138]. Future work should focus on transferring the findings on contextual factors such as interruptibility or boredom by using the day of the week [138], time of day [138], location [137], or different proxies for social context (eg, number of calls and messages sent or received) [138] to investigate tailoring to the state of receptivity.

Taken together, these findings highlight that although the JITAI concept seems to be widely known in the scientific digital health community [24] and different studies outline the possibility of detecting changes in depression or *depressive symptoms* such as mood by using different passive measurements [121,126-132], these mechanisms have not been implemented in the real world, aside from baseline or progress assessments. Although tailoring to both the state of vulnerability and state of receptivity may be challenging, it is still surprising that no JITAI mechanisms have been implemented by the reviewed apps. This finding is especially unexpected given the existing work on passive sensing and the popularity of the JITAI concept.

Related to these findings, we were interested in the degree to which the effectiveness and efficacy of the included apps were investigated in peer-reviewed publications because it has been proposed that JITAIs increase the effectiveness or efficacy of such apps. None of the 70 reviewed publications investigated JITAI mechanisms. Therefore, our findings highlight that the proposed increase in effectiveness or efficacy achieved by using JITAIs is not evaluated in real-world app settings. In addition, we found great variability in the scientific evidence provided for the reviewed apps—despite an increased interest in digital health—in several publications addressing this topic, especially within the last 5 years [26,139,140].

### Limitations

The strengths of this study are the large number of apps initially screened; the analyses using a framework developed from existing work; and the rigorous methodology used to review all identified studies addressing the apps, the apps’ websites, and the apps themselves. However, this study includes several limitations. We reviewed the apps at a single point, which is a shortcoming found in related studies as well. We are aware that the app stores are dynamic with constant changes [25], but a long-term review of the apps would not have been feasible. We may address this issue in our future work. Besides the lists we reviewed from the Anxiety and Depression Association of America, National Health Service, and American Psychological Association, rating systems or lists of reviewed apps are offered by other organizations, too. These include, but are not limited to, the American Psychiatric Association, PsyberGuide, and iMedicalApps. We did not review these lists because we expected a high number of overlaps and because not all the apps found in the lists had been reviewed (eg, Dartmouth PATH had not been reviewed on PsyberGuide when last checked [January 27, 2021]), and not all the apps mentioned in the lists had been recommended (eg, Mood Watch Review, with low credibility, user experience, and transparency ratings on PsyberGuide when last checked [January 27, 2021]). However, we see the value in a central platform for reviews of mental health apps and suggest incorporating the findings regarding the use of JITAI mechanisms into the existing review criteria.

We did not search for apps developed by research groups as was done in a recent meta-analytic review by Wang and Miller [24] that evaluated studies addressing the effectiveness of JITAIs. Their search included, but was not limited to, apps. Although this work is highly relevant for the field, we argue that a review investigating the degree to which tailoring is used in popular apps was necessary. This notion was derived from findings highlighting that academia has not always kept up with the high speed of digital health developments in the real world [41]. In addition, apps developed for research purposes may not be available to the public, limiting the impact of these apps to a small number of individuals participating in the review. Our aim was to investigate whether popular apps that reach many individuals use these promising mechanisms. Finally, the review of the apps initially included other aspects such as the use of evidence-based treatment, conversational agents, and the revenue model. Reporting these findings would have exceeded the scope of this review.

### Comparison With Prior Work

We found 11 reviews investigating different aspects of the apps that address depression [14,20,26,27,29,141-145]. Of these 11 reviews, 6 (45%) assessed the content or features of the apps [14,20,26,29,141,145]; of these 6 reviews, 1 (17%) adjusted its analysis to the number of users [27]. A meta-analysis focused on the efficacy of apps for depression, indicating a moderate positive effect when the apps were compared with inactive control and a small effect when comparing the apps with active control [20]. The remaining studies investigated usability [143], adherence to clinical guidelines [144], claims [142], or data sharing and privacy practice [142]. A meta-analytic review investigated the effect sizes of JITAIs compared with control groups or other interventions, but it did not focus on apps or mental health [24]. This meta-analytic review indicated that two aspects of tailoring—*tailoring to what* and the tailoring *approach*—were significantly associated with greater JITAI efficacy. We found no study investigating the use of JITAI mechanisms or reviewing the measurements used to capture changes when discussing the relevant features of such apps. Furthermore, we did not find any studies reviewing whether real-world apps provide evidence of an improvement in their effectiveness or efficacy achieved by using JITAI mechanisms.

### Conclusions

In conclusion, our findings indicate that because of the limited use of measurements for depressive symptoms, except for self-reports as indicators of progress or initial tailoring, the 28 most popular or recommended apps that address depression cannot be considered JITAIs. Although self-reports and, occasionally, passive measurements were used to capture changes in depressive symptoms, these changes were not used to dynamically tailor the content to a state of vulnerability or state of receptivity. An increase in app effectiveness or efficacy achieved by using JITAI mechanisms was also not evaluated in any of the reviewed publications. Therefore, future work should focus on evaluating the effectiveness of real-world JITAIs that tailor their content and timing to the state of vulnerability and state of receptivity. On the basis of these findings, we argue that the reviewed apps do not yet leverage the full potential of digital health interventions by providing tailored support when it is most needed and in the most helpful way.

## Appendix

Multimedia Appendix 1 Codebook for app review, calculations of downloads and reviews covered, number of studies and type of studies found for each app, and list of all reviewed publications.

## Abbreviations

JITAI: just-in-time adaptive intervention
