# Supplementary material for: Just-in-Time Adaptive Mechanisms of Popular Mobile Apps for Individuals With Depression: Systematic App Search and Literature Review
Source: J Med Internet Res. 2021 Sep 28;23(9):e29412. doi: 10.2196/29412 (PMC8512178; doi:10.2196/29412)
Supplement: Multimedia Appendix 1 [file jmir_v23i9e29412_app1.docx]

## Review

# Just-in-time Adaptive Mechanisms of Popular Mobile Applications for Individuals with Depression: Systematic Review

# Supplementary Files

**Table S1.** Codebook along which the apps were rated.

| **Code** | **Question** | **Source** | **Description** |
| --- | --- | --- | --- |
| G1 | Name | - | Name App |
| G2 | Provider/ Company | - | Name Provider/ Company |
| G3 | Affiliated Organizations | - | Affiliated Organizations (Companies, Universities, Governments, NGOs) |
| G4 | Websites | - | Intervention's Website, Company´s Website |
| G5 | Google Play Store Information | - | Google Play Store: Number Downloads, Number Reviews, Average Review, Last updated |
| G6 | Apple App Store Information | - | Apple App Store: Number Reviews, Average Review, Last updated |
| G7 | Prescription or Access Code | - | Is a Prescription, Access Code Necessary |
| J1 | How are the states of vulnerability measured? | Nahum-Shani et al (2018)[21] | Codes used are DF1-12 for Symptoms, PS1-12 for Sensors, DA1-10 Device Analytics, and SR1-11 for Self-Reports. |
| J4 | How are distal outcomes measured? | Nahum-Shani et al (2018)[21] | Codes used are DF1-12 for Symptoms, PS1-12 for Sensors, DA1-10 Device Analytics, and SR1-11 for Self-Reports. |
| J5 | How are proximal outcomes measured? | Nahum-Shani et al (2018)[21] | Codes used are DF1-12 for Symptoms, PS1-12 for Sensors, DA1-10 Device Analytics, and SR1-11 for Self-Reports. |
| J8 | How are tailoring variables measured? | Nahum-Shani et al (2018)[21] | Codes used are DF1-12 for Symptoms, PS1-12 for Sensors, DA1-10 Device Analytics, and SR1-11 for Self-Reports. |
| E1 | Is the intervention compared to a gold standard for treating depression? | Mathews et al (2019)[41] | Yes or no used and short description to what it is compared to. |
| E2 | Is the intervention compared to other care quality metrics such as measures of clinical outcome? | Mathews et al (2019)[41] | Yes or no used and short description to what it is compared to. |
| E3 | Are academics and/or clinicians involved? If yes, how? | Mathews et al (2019)[41] | Yes or no and short description of how they were involved. |
| E4 | Description of each study | Safavi et al (2019)[38] | Each study was evaluated against EV1-11. |

**Table S2.** Codes for the classification of depression symptoms.

| **Code** | **Symptom** | **Source** | **Description** |
| --- | --- | --- | --- |
| DF1 | Activity | DSM-V, ICD-10 | Fatigue, loss of energy or activity, psychomotor agitation or retardation |
| DF2 | Mood | DSM-V, ICD-10 | Depressive mood, loneliness, or sadness |
| DF3 | Interest/Pleasure | DSM-V, ICD-10 | Diminished interest or loss of pleasure |
| DF4 | Weight | DSM-V | Weight loss or weight gain |
| DF5 | Appetite | ICD-10 | Poor or increased appetite |
| DF6 | Cognition | DSM-V | Diminished cognitive performance or indecisiveness |
| DF7 | Unhelpful Beliefs | DSM-V, ICD-10 | Feelings of inappropriate guilt worthlessness, or loss of confidence |
| DF8 | Concentration | DSM-V, ICD-10 | Diminished ability to concentrate |
| DF9 | Suicidal Thoughts | DSM-V | Recurrent thoughts of death or recurrent suicidal ideation |
| DF10 | Sleep | ICD-10 | Insomnia or hypersomnia |
| DF11 | Illogical Thinking | Barlow et al (2017)[42] | Deriving at an illogical conclusion or biased attributions |
| DF12 | Distorted perception | Barlow et al (2017)[42] | Focus on negative experiences or thoughts |
| DF13 | Demographics | ·· | Questions about demographics such as age and gender |
| DF14 | Social Economic Status | ·· | Questions about e.g. education, income, and social status |

**Table S3.** Codes for the classification of self-reports.

| **Code** | **Self-Reports** | **Source** | **Description** |
| --- | --- | --- | --- |
| SR1 | Open Questions | ·· | Questions with non-restricted input |
| SR2 | Ratings | ·· | Questions answered with a scale (e.g. Likert Scale) |
| SR3 | Multiple Choice | ·· | Question with a given number of answer options |
| SR4 | PHQ | Kroenke et al (2002)[43] | Patient Health Questionnaire |
| SR5 | HDI | Hamilton et al (1986)[44] | Hamilton Depression Inventory |
| SR6 | M.I.N.I. 5.0 | Sheehan et al (1998)[45] | Mini-International Neuropsychiatric Interview |
| SR7 | BDI-II | Beck et al (19 96)[46] | Beck Depression Inventory II |
| SR8 | OQ-45 | Lambert et al (2004)[47] | The Outcome Questionnaire-45 |
| SR9 | CORE-OM | Evans et al (2000)[48] | The CORE Outcome Measurement |

**Table S4.** Codes for the classification of sensors.

| **Code** | **Sensor** | **Source** | **Description** |
| --- | --- | --- | --- |
| PS1 | Vital Signs | Android Developers Guide (2020)[39] | The app can access sensor data about vital signs |
| PS2 | Location | Android Developers Guide (2020)[39] | The app can access the device's location |
| PS3 | Physical Activity | Android Developers Guide (2020)[39] | The app can access recordings of physical activity, such as walking, cycling, driving, step count, etc. |
| PS4 | Camera | Android Developers Guide (2020)[39], iOS Security Guide(2020)[40] | The app can take pictures and record video |
| PS5 | Microphone | Android Developers Guide (2020)[39], iOS Security Guide (2020)[40] | The app can record audio |
| PS6 | Speech recognition | Android Developers Guide (2020)[39], iOS Security Guide (2020)[40] | The app can use speech recognition |
| PS7 | Bluetooth | Android Developers Guide (2020)[39], iOS Security Guide (2020)[40], Cornet et al (2018)[23] | The app can detect or communicate with other Bluetooth-enabled devices |
| PS8 | Accelerometer | Cornet et al (2018)[23] | The app can measure the speed of movement in space and the speed of the rotation of the device |
| PS9 | GPS | Cornet et al (2018)[23] | The app can use the information of four or more GPS satellites to calculate the position of the device |
| PS10 | Antenna | Cornet et al (2018)[23] | The app can use the information of nearby cellular towers and relay the single to the broadband processor for voice/SMS/ data communication |
| PS11 | Light Sensor | Cornet et al (2018)[23] | The app can measure the amount of light reaching the device |
| PS12 | Proximity | Cornet et al (2018)[23] | The app can detect the proximity between the front of the phone and any obstacle, such as a human face |

**Table S5.** Codes for the classification of device analytics.

| **Code** | **Device Analytics** | **Source** | **Description** |
| --- | --- | --- | --- |
| DA1 | Storage | Android Developers Guide (2020)[39] | The app can access photos, media, and files on the device |
| DA2 | Telephone | Android Developers Guide (2020)[39] | The app can make and manage phone calls |
| DA3 | Photos | Android Developers Guide (2020)[39] | The app can access the photos stored |
| DA4 | Contacts | Android Developers Guide (2020)[39], iOS Security Guide (2020)[40] | The app can access the phone contacts |
| DA5 | Call logs | Android Developers Guide (2020)[39], Cornet et al (2018)[23] | The app can access the number of times a call was made, accepted, and missed |
| DA6 | Calendar | Android Developers Guide (2020)[39], iOS Security Guide (2020)[40] | The app can access the calendar |
| DA7 | Device activity | Cornet et al (2018)[23] | The app can access recorded activity time of the device |
| DA8 | SMS patterns | Android Developers Guide (2020)[39], Cornet et al (2018)[23] | The app can access the number of messages send and received |
| DA9 | Application usage | Cornet et al (2018)[23] | The app can access the apps used and times the apps were used |
| DA10 | Browser history | Cornet et al (2018)[23] | The app can access visited websites |

**Table S6.** Codes for the extraction of information about each of the reviewed publications.

| **Code** | **Study Information** | **Source** | **Description** |
| --- | --- | --- | --- |
| EV1 | Publication Year | Safavi et al (2019)[38] | Year of publication |
| EV2 | Journal Name | Safavi et al (2019)[38] | Name of journal |
| EV3 | Journal Impact factor | Safavi et al (2019)[38] | Impact factor of journal (if available 5 years impact factor) |
| EV4 | Number Subjects | Safavi et al (2019)[38] | Total number of subjects (control and treatment combined) |
| EV7 | Study Purpose | Safavi et al (2019)[38] | Area investigated by paper |
| EV8 | Type of Study | ·· | Design used (e.g. RCT, parallel group, lab-experiment) |
| EV 8 | Provides Information About | ·· | JITAI concept, conversational agent, effectiveness, efficacy, feasibility, etc. |
| EV9 | Measurements Used | ·· | Measurements used to describe changes or outcomes |
| EV10 | Measuring Depression | ·· | Symptoms of depression measured by validated instruments such as questionnaires or clinical interviews |
| EV11 | Comparison to other treatment | ·· | Was the app compared to standard treatment such as face-to-face therapy or medication |

**Table S7.** All apps that were found and screened with source from which they were found, decision to include (I), exclude (E), or duplicate (d) and reason for the decision.

| **App** | **Source** | **Down-load Category** | **Avr. Reviews** | **Re-views** | **Decision** | **Reason** | **Details** |
| --- | --- | --- | --- | --- | --- | --- | --- |
| #Reasons2 | IOS | 0 | 4.3 | 7 | E | Focus not depression | General mental health focus |
| #SelfCare | AND | 500000 | 4.3 | 7071 | I | - | - |
| 10 Daily Stress Relief Yoga and Exercise | AND | 1000 | 4.7 | 27 | I | - | - |
| 3D Brain | IOS | 0 | 4.1 | 73 | D | D | D |
| 3D Brain | APA | 0 | 4.1 | 73 | E | Focus not depression | Information about the brain |
| 500+ quotes for every mood | AND | 500000 | 4.5 | 2732 | E | Focus not depression | Providing quotes for mood |
| 7 Cups: Anxiety & Stress Chat | IOS/AND | 1000000 | 3.5 | 17021 | I | - | - |
| 7Mind: Meditation reinvented | AND | 500000 | 4.7 | 8255 | E | Focus not depression | Meditation without a specific mental health or depression focus |
| A Day in the Life - Living With Depression | AND | 50 | 1 | 1 | I | - | - |
| ACR Digital Urinalysis | NHS |  |  |  | E | Focus not depression | Focus on urinalysis |
| ACT iCoach | AND | 5000 | 4.8 | 604 | E | Focus not depression | ACT therapy but not mentioning depression |
| Action For Happiness | IOS | 0 | 4.9 | 68 | E | Focus not depression | Focus on happiness and not depression |
| Affirm Me: Daily Affirmations | IOS | 0 | 4.8 | 69 | E | Focus not depression | Providing daily affirmation |
| All Mental Disorders and Treatment | IOS/AND | 50000 | 4.3 | 1229 | I | - | - |
| Am I Depressed? | AND | 10 | NA | NA | E | No active component | Depression triage without any intervention component |
| Anger Management & stress relief game (pstd) | AND | 50000 | 2.8 | 351 | E | No active component | Collection of games with no therapeutic content |
| Anger Management Tips! | IOS | 0 | 4.3 | 25 | E | Focus not depression | Anger management not depression |
| Anti-Depression Diet - 3 to 7 Days | AND | 500 | NA | NA | I | - | - |
| Antistress ASMR Relaxing Games | IOS | 0 | 4.7 | 90 | E | Focus not depression | Antistress not depression |
| AntiStress, Relaxing, Anxiety & Stress Relief Game | IOS/AND | 1000000 | 3.6 | 20386 | E | Focus not depression | Stress and anxiety focus not depression |
| Anxiety & Depression Symptoms - Millionaire Mind | AND | 1000 | 3.9 | 7 | E | No active component | Information about depression |
| Anxiety & Depression Symptoms - Twayesh Project | AND | 100000 | 3.3 | 317 | E | No active component | Information about depression |
| Anxiety Coaches Podcasts & Workshops by Gina Ryan | IOS | 0 | 5 | 6 | E | Focus not depression | Focus on anxiety and not depression |
| Anxiety No More: Strategies for Anxiety, Depression, and Panic | IOS | 0 | 4.1 | 28 | E | Focus not depression | Focus on anxiety and not depression ("tackling generalized anxiety disorder, social anxiety disorder, panic disorder, and post-traumatic stress disorder") |
| Anxiety Quotes | AND | 1000 | 4.5 | 13 | E | No active component | Providing quotes |
| Anxiety Relief Hypnosis - Stress, Panic Attacks | AND | 100000 | 4.3 | 1362 | E | Focus not depression | Focus on anxiety and not depression |
| Anxiety relief journal: mental health & depression | AND | 10000 | 4.9 | 425 | I | - |  |
| Anxiety Reliever | ADAA |  |  |  | E | Focus not depression | Focus on anxiety and not depression |
| Anxiety Test | IOS | 0 | NA | NA | E | Focus not depression | Focus on anxiety and not depression |
| Anxiety Test GAD-7 | IOS | 0 | 4.7 | 69 | E | Focus not depression | Focus on anxiety and not depression |
| Anxiety Tracker - Stress and Anxiety Log | AND | 10000 | 4.4 | 214 | E | Focus not depression | Focus on anxiety and not depression |
| AnxietyCoach | ADAA |  |  |  | E | Focus not depression | Focus on anxiety and not depression |
| anxietyhelper | IOS | 0 | 4.4 | 94 | E | Focus not depression | Focus on anxiety and not depression |
| Atlas Co | IOS | 0 | 4.9 | 108 | E | Focus not depression | Private social network to share with closes friends to build connection |
| Audiojoy for Self-Improvement | IOS | 0 | 4.6 | 29 | E | Focus not depression | Focus on self-improvement not depression |
| Aurum - stress, anxiety, self-help, therapy. | AND | 10000 | 4.5 | 290 | I | - |  |
| Baby and Child First Aid | NHS |  |  |  | E | Focus not depression | Focus on children health |
| Baby Buddy | NHS | 10000 | 4.5 | 290 | E | Focus not depression | Focus on children health |
| Battlefield of the Mind Devotional | IOS | 0 | 4.9 | 119 | E | Focus not depression | Focus on theology and bible quotes |
| Be Mindful | AND | 0 | 0 | 0 | D | D |  |
| Be Mindful | NHS | 0 | 0 | 0 | E | No access | No access |
| Bearable - Mood & Symptom tracker (BETA) | AND | 5000 | 4.9 | 69 | I | - |  |
| Beat depression | AND | 10000 | 4.2 | 301 | I | - |  |
| Beat Panic | NHS |  |  |  | E | Focus not depression | Focus on panic disorder not depression |
| Becca Breast Cancer Support App | NHS | 10000 | 4.2 | 301 | E | Focus not depression | Focus on breast cancer not depression |
| Behavioral Psychology | IOS/AND | 1000 | 4 | 34 | I | - |  |
| Better app | IOS | 0 | 5 | 15 | I | - |  |
| Better Stop Suicide | IOS | 0 | 4.7 | 182 | E | Focus not depression | Focus on suicide not depression |
| BetterHelp: Online Counseling & Therapy | IOS/AND | 500000 | 4.5 | 9302 | I | - |  |
| Beyond Meditation | IOS | 0 | 4.3 | 42 | E | Focus not depression | Meditation without a specific mental health or depression focus |
| Big White Wall | NHS | 0 | 0 | 0 | E | No access | No access |
| Binaural Beats Machine | IOS | 0 | 4.6 | 43 | E | Focus not depression | Focus on sounds not depression |
| Binaural Beats Therapy | AND | 1000000 | 4.4 | 14291 | E | Focus not depression | Focus on sounds not depression |
| Bipolar Disorder Test | IOS | 0 | 4.6 | 59 | E | Focus not depression | Focus on bipolar disorder not depression |
| Bipolar test - Baris Sarer | IOS | 0 | 4.4 | 14 | E | Focus not depression | Focus on bipolar disorder not depression |
| Bipolar test - MoodTools | AND | 10000 | 3.1 | 35 | E | Focus not depression | Focus on bipolar disorder not depression |
| Bird Sounds, Listen & Relax | IOS | 0 | 4.5 | 1855 | E | Focus not depression | Focus on sounds not depression |
| Bloom: CBT Therapy & Self-Care | IOS | 0 | 4.8 | 67 | I | - |  |
| BlueIce | NHS |  |  |  | E | Focus not depression | Focus on self-harm |
| Booster buddy | IOS/AND | 100000 | 4.3 | 1469 | I | - |  |
| Braineka Depression | AND | 10 | NA | NA | I | - |  |
| BrainSights - Neuroscience | IOS | 0 | 4.9 | 48 | E | Focus not depression | Information about the brain |
| BrainwaveX Depression | AND | 50 | NA | NA | I | - |  |
| BrainwaveX Depression Pro | IOS | 0 | NA | NA | I | - |  |
| Breathe Get Energy & Depression Help by Calming Music, Sounds mixer | IOS | 0 | 5 | 4 | I | - |  |
| Breathe: Stress Relief Meditation | AND | 10000 | 4.6 | 123 | I | - |  |
| Breathe2Relax | ADAA |  |  |  | D | D | Cancer |
| Breathe2Relax | APA |  |  |  | E | Focus not depression | Focus on stress-relief not depression |
| Breathwrk: Breathing Exercises | IOS | 0 | 4.9 | 1323 | E | Focus not depression | Focus on stress-relief not depression |
| Breeze: mood tracker, diary | IOS | 0 | 4.6 | 1153 | I | - |  |
| Brush DJ | NHS |  |  |  | E | Focus not depression | Focus on children dental health |
| Calm - meditate, sleep, relax | AND | 10000000 | 4.4 | 248387 | I | - |  |
| Calm Harm - manages self-harm | AND | 100000 | 4.3 | 1573 | E | Focus not depression | Focus on self-harm |
| Carpe Diem - Depression and Anxiety Forum | AND | 1000 | 4.9 | 98 | I | - |  |
| Catch It | NHS |  |  |  | I | - | Cancer |
| Catch It - Make sense of moods | IOS/AND | 0 | 2.5 | 31 | D | D |  |
| CBT Companion: (Cognitive Behavioral Therapy app) | AND | 10000 | 5 | 870 | I | - |  |
| CBT Diary | AND | 10000 | 4.2 | 239 | I | - |  |
| CBT Thought Diary | IOS | 0 | 4.8 | 481 | I | - |  |
| CBT Thought Diary - Mood Tracker, Journal & Record | AND | 100000 | 4.6 | 1217 | I | - |  |
| CBT Tools for Healthy Living, Self-help Mood Diary | AND | 100000 | 4.3 | 1660 | I | - |  |
| CBT-I | APA |  |  |  | E | Focus not depression | Focus on veterans and insomnia |
| Celebrate Recovery | IOS | 0 | 4.4 | 17 | E | Focus not depression | Focus on biblical and balanced program |
| CESD depression test | AND | 10000 | 4.4 | 122 | E | No active component | Depression triage without any intervention components |
| Changing Health | NHS |  |  |  | E | Focus not depression | Focus on general health and lifestyle |
| ChatHealth | NHS |  |  |  | E | Focus not depression | Focus on general health and lifestyle |
| Chill Panda | NHS | 10000 | 4.4 | 122 | E | Focus not depression | Focus on children relaxation |
| Chromatic: Color Puzzles | IOS | 0 | 4.8 | 71 | E | Focus not depression | Stress reducing game |
| Clinical depression | AND | 1000 | NA | NA | E | No active component | Providing quotes |
| Co-op Health | NHS |  |  |  | E | Focus not depression | NHS medication ordering |
| Coach Me 2 be Calm Mindfulness | IOS | 0 | 4.5 | 62 | E | Focus not depression | Focus on stress-relief not depression |
| Cognitive Behavioral Therapy - CBT | AND | 10000 | 3.4 | 178 | I | - |  |
| Coloring Book Swear Words - For ADULTS | IOS | 0 | 4.1 | 597 | E | Focus not depression | Stress reducing game |
| Colorjoy: Coloring Book for Adults and Kids | IOS | 0 | 4.4 | 625 | E | Focus not depression | Stress reducing game |
| Combined Minds | IOS | 0 | 5 | 10 | E | Focus not depression | Focusing on young person mental health condition |
| Complete Relaxation: Lite | IOS | 0 | 4.8 | 224 | E | Focus not depression | Stress and relaxation not depression |
| Control and Monitor: Anxiety, Mood and Self-Esteem | AND | 1000000 | 4.6 | 15129 | I | - |  |
| Copeify, Self-Care @Scale | IOS | 0 | 5 | 57 | E | No active component | Depression triage without any intervention components |
| Corona-Help.UK | NHS |  |  |  | E | Focus not depression | General mental health focus with focus on corona |
| Counseling - Talk, Chat & Video Conference - Live | AND | 5000 | 4.4 | 112 | I | - |  |
| Cove | NHS |  |  |  | D | D | Mental health |
| Cove: music for mental health | IOS | 0 | 4.7 | 284 | E | Focus not depression | Focus on sounds not depression |
| Cover art Stress Control Norbu - meditation, relaxing games | IOS/AND | 10000 | 4.3 | 111 | I | - |  |
| CoVerse - Advice and Chat | AND | 5000 | 4.7 | 370 | E | Focus not depression | Focus on general mental health and social platform for unbiased advice or opinions |
| CPT Coach | ADAA |  |  |  | E | Focus not depression | Focus on general mental health and not depression |
| Cure Depression & Anxiety with Breath | AND | 100 | 4.6 | 45 | I | - |  |
| Daily Affirmations - Fill your day with positivity | AND | 100000 | 4.6 | 1760 | E | Focus not depression | Focus on general mental health and not depression |
| Daily Affirmations! Self Confidence Improvement and Unique Positive Thinking Companion FREE! | IOS | 0 | 4.6 | 233 | E | Focus not depression | Focus on general mental health and not depression |
| Daily Feats | AND | 100 | 5 | 2 | E | Focus not depression | Focus on daily accomplishments and not depression |
| Daily Inspirational Quotes Pro | IOS | 0 | 4.6 | 187 | E | No active component | Providing quotes |
| Daily Quotes: quote of the day | IOS | 0 | 4.4 | 334 | E | No active component | Providing quotes |
| Daily Tracker+ Journal & Diary | IOS | 0 | 4.5 | 1708 | E | Focus not depression | Activity tracker not depression |
| Dare - Break Free from Anxiety | AND | 100000 | 4.8 | 2808 | E | Focus not depression | Focus on anxiety and not depression |
| Darry - daily mood & journal | IOS | 0 | 3.9 | 7 | I | - |  |
| Day to Day | AND | 100 | 5 | 3 | E | Focus not depression | Focus on skill development and not depression |
| Daylio - Diary, Journal, Mood Tracker | AND | 5000000 | 4.7 | 292548 | I | - |  |
| DBT Coach | IOS | 0 | 4.7 | 514 | I | - |  |
| DBT Diary Card & Skills Coach | IOS | 0 | 4.6 | 251 | E | Focus not depression | Focus on dialectical behavior therapy but not focused on depression |
| DBT Trivia & Quiz | IOS | 0 | 4.3 | 233 | E | Focus not depression | Focus on dialectical behavior therapy but not focused on depression and quiz information not components |
| Deal with Depression Offline Guide | IOS | 1 | 5 | 1 | I | - |  |
| Dealing with depression | IOS/AND | 5000 | 4.5 | 97 | I | - |  |
| Dealing with Depression Guide | AND | 100 | NA | NA | I | - |  |
| Dealing with Depression, Depression Treatment | IOS/AND | 50 | NA | NA | I | - |  |
| Deep Sleep, Insomnia Help | IOS | 0 | 4.5 | 154 | E | Focus not depression | Focus on insomnia not depression |
| Depression - AppCounselor | AND | 500 | 3.7 | 7 | E | No active component | Depression triage without any intervention components |
| Depression - Doctot | IOS | 0 | NA | NA | D | D |  |
| Depression - Doctot | IOS | 0 | NA | NA | E | No active component | Depression triage without any intervention components |
| Depression - EL MAKAOUI | AND | 10 | NA | NA | E | No active component | Information about depression |
| Depression - Focus Medica India Pvt. Ltd | AND | 100 | NA | NA | E | No active component | Information about depression |
| depression - Free Entertainment apps | AND | 1000 | 3 | 5 | E | No active component | Information about depression |
| Depression - Mental Health Awareness | IOS/AND | 100 | 5 | 1 | I | - |  |
| Depression - MMI | AND | 50 | NA | NA | E | No access | No access |
| Depression - Mukesh Meena | AND | 1000 | 4.8 | 30 | E | Not English | Not English |
| Depression - nodepression | IOS | 0 | NA | NA | E | No active component | Information about depression |
| Depression - Personal remedies LLC | IOS | 0 | NA | NA | E | No active component | Food information for a range of mental health problems |
| Depression & Anxiety Magazine | IOS | 0 | NA | NA | E | No access | No access |
| Depression & Anxiety Self-Test (Africa's Version) | IOS/AND | 1000 | 3.2 | 6 | E | No active component | Depression triage without any intervention components |
| Depression & Bipolar Disorder | IOS/AND | 5000 | 4.5 | 15 | I | - |  |
| Depression and Psychology by GoLearningBus | IOS | 0 | NA | NA | E | Aimed at professionals | Teaching Tool for University |
| Depression app | AND | 1000 | 4 | 30 | E | No active component | Depression triage without any intervention components |
| Depression Awareness | IOS/AND | 500 | 5 | 1 | E | Focus not depression | Information about depression |
| Depression Calculator | IOS | 0 | NA | NA | E | No active component | Depression triage without any intervention components |
| Depression CBT Self-Help Guide | IOS/AND | 100000 | 4.2 | 1448 | I | - |  |
| Depression Cure Music | AND | 50000 | 4.5 | 561 | I | - |  |
| Depression Door Karne Ke 90 Upay In Hindi | AND | 100 | 3 | 1 | E | Not English | Not English |
| Depression Guide | AND | 10 | 9 | 5 | E | No active component | Depression triage without any intervention components |
| Depression Help | AND | 10 | NA | NA | I | - |  |
| Depression Journal | IOS | 0 | NA | NA | I | - |  |
| Depression Manager | IOS | 0 | NA | NA | I | - |  |
| Depression Medication Manager (Early Access) | AND | 1000 | NA | NA | I | - |  |
| Depression Monitor | AND | 500 | NA | NA | I | - |  |
| Depression Natural Treatment | AND | 500 | NA | NA | E | No active component | Information about depression |
| Depression Psychopharmacology | IOS | 0 | NA | NA | E | Aimed at professionals | Aimed at professionals |
| Depression Quote Wallpapers - ARD Studio Dev | IOS/AND | 100 | NA | NA | E | No active component | Providing quotes |
| Depression Quote Wallpapers - HD Wallpapers Pro | AND | 10 | NA | NA | E | No active component | Providing quotes |
| Depression Quote Wallpapers - Leafgreen | AND | 100000 | 4.5 | 1506 | E | No active component | Providing quotes |
| Depression Quote Wallpapers - Modux Apps | AND | 100000 | 4.4 | 540 | E | No active component | Providing quotes |
| Depression Quote Wallpapers - Wallpapers appx | AND | 10 | NA | NA | E | No active component | Providing quotes |
| Depression Quote Wallpapers - Wishingeveryone | AND | 10 | 5 | 1 | E | No active component | Providing quotes |
| Depression Quote Wallpapers - Zexica Apps | AND | 500 | NA | NA | E | No active component | Providing quotes |
| Depression Quote Wallpapers HD - App Makerz | AND | 1000 | 4.3 | 6 | E | No active component | Providing quotes |
| Depression quotes - Angelworls | AND | 10000 | 4.5 | 63 | E | Focus not depression | Providing quotes |
| Depression quotes - Dub Apps | AND | 1000 | 4 | 12 | E | No active component | Providing quotes |
| Depression quotes - FrozenWaveApps | AND | 10000 | 2.8 | 53 | E | Focus not depression | Providing quotes |
| Depression quotes - Knowledge Project | AND | 500 | 3 | 3 | E | No active component | Providing quotes |
| Depression Quotes - Nerd Pig | AND | 500 | NA | NA | E | No active component | Providing quotes |
| Depression Quotes - ShinelikeDocras | AND | 50 | NA | NA | E | No active component | Providing quotes |
| Depression Quotes Offline - Phone Wallpapers | AND | 10 | NA | NA | E | No active component | Providing quotes |
| Depression Quotes Wallpaper 😞 - High D Wallpaper | AND | 1000 | 4 | 8 | E | No active component | Providing quotes |
| Depression Quotes Wallpaper HD - Daily Hot Free Apps | AND | 10000 | 4.6 | 78 | E | No active component | Providing quotes |
| Depression Quotes: Collection of Depressed Quotes for Sad Moments - BearTech Bilisim | IOS | 0 | 3.5 | 11 | E | No active component | Providing quotes |
| Depression Quotes: Sadness and Pain Quotes - Marble Apps | AND | 10000 | 4 | 100 | E | No active component | Providing quotes |
| Depression relief Help of self-hypnosis subliminal - Alisa Unzhakova | IOS | 0 | 5 | 1 | E | No active component | Providing quotes |
| Depression screening tool: PHQ-9 test - Psycnet Software | AND | 100 | 5 | 4 | E | No active component | Depression triage without any intervention components |
| Depression Self Help Guide: CBT - Xandy App Ideas | AND | 5000 | 3.6 | 12 | E | No active component | Depression triage without any intervention components |
| Depression support - MyHealthteams | AND | 1000 | 2.9 | 39 | I | - |  |
| Depression Test - Am I Depressed Personality Test - Marko Petkovic | IOS | 0 | NA | NA | E | Focus not depression | Personality test and not depression |
| Depression Test - Renu Bala HAU | AND | 500 | 4.5 | 6 | E | No active component | Depression triage without any intervention components |
| Depression Test & Help To Fight Depression - Awesome App Ideas | IOS | 5 | NA | NA | E | No active component | Depression triage without any intervention components |
| Depression Test \| PHQ-9 - Eddie Liu | IOS | 0 | 4.6 | 67 | E | No active component | Depression triage without any intervention components |
| Depression Test and Training - CogniFit | IOS | 0 | 5 | 2 | E | No active component | Depression triage without any intervention components |
| Depression Test By Pocket Shrink - PocketShrink | IOS | 0 | 5 | 1 | E | No active component | Depression triage without any intervention components |
| Depression Test - Androbot Tech | AND | 500 | 4.1 | 10 | E | No active component | Depression triage without any intervention components |
| Depression Test - Barsis Sarer | AND | 5000 | 4.3 | 31 | E | No active component | Depression triage without any intervention components |
| Depression Test - Delight Dev | AND | 10 | 5 | 1 | E | No active component | Depression triage without any intervention components |
| Depression Test - Eyyup Alkis | AND | 500 | 4.4 | 14 | E | No active component | Depression triage without any intervention components |
| Depression Test - FXT Tech | AND | 1000 | 3 | 12 | E | No active component | Depression triage without any intervention components |
| Depression Test - Japps Medical | AND | 100000 | 3.8 | 1487 | E | No active component | Depression triage without any intervention components |
| Depression Test - Mood Tools | AND | 100000 | 3.9 | 418 | E | No active component | Depression triage without any intervention components |
| Depression Test - Progressive Programming | AND | 50000 | 4.6 | 1094 | E | No active component | Depression triage without any intervention components |
| Depression Treatment - Knowledge and Tips - TUTORIFY | IOS/AND | 100 | NA | NA | E | No active component | Information about depression |
| Depression Treatment help by Yoga, Exercise & Diet-Dr. Zio - Yoga teacher | AND | 100 | NA | NA | I | - |  |
| DEPRESSION TREATMENT - Supportive Apps | IOS/AND | 1000 | 5 | 1 | I | - |  |
| Depression Wallpapers - Atlas Labs | IOS | 0 | 5 | 4 | E | No active component | Providing quotes |
| Depression Wallpapers - wallpapers and background | AND | 5000 | 4.1 | 26 | I | - |  |
| Depression ZX | IOS | 0 | 5 | 6 | I | - |  |
| Depression: causes, symptoms & treatment | IOS/AND | 100 | NA | NA | E | Focus not depression | Information about depression |
| Depression: The Game | AND | 10000 | 3.6 | 224 | I | - |  |
| Depressive and sad wallpaper | IOS/AND | 50000 | 4.2 | 350 | E | Focus not depression | Providing quotes |
| DeStressify Stress Relief | IOS | 0 | NA | NA | E | Focus not depression | Focus on stress-relief not depression |
| Digital Health Passport | NHS |  |  |  | E | Focus not depression | Collection of digital health information |
| distrACT | NHS |  |  |  | E | Focus not depression | Focus on self-harm |
| Doctor On Demand | IOS | 0 | 4.9 | 59894 | E | Focus not depression | Video Consultation |
| Don't panic - Depression and panic help | AND | 50000 | 3.8 | 460 | I | - |  |
| Dreamfora: Dream, Habit, Task & Daily Motivation | AND | 100000 | 4.4 | 845 | E | Focus not depression | Focus on habits and motivation |
| DrJulian | IOS | 0 | NA | NA | I | - |  |
| DropBox | APA |  |  |  | E | Focus not depression | File sharing platform (recommended by APA for professionals) |
| DRT | IOS | 0 | 4.8 | 37 | E | Focus not depression | Physical Activity App |
| Easy Mood Diary | IOS | 0 | 3.7 | 22 | I | - |  |
| Echo Pharmacy | NHS |  |  |  | E | Focus not depression | Ordering medication |
| Edinburgh Postnatal Depression Scale (EPDS) | AND | 10 | NA | NA | E | No active component | Depression triage without any intervention components |
| Elevate \| Inspirational Living | IOS | 0 | 4.9 | 48 | E | Focus not depression | Focus on inspirational living tips |
| Ellie: Meet Disabled Singles | IOS | 0 | 3.9 | 17 | E | Focus not depression | Dating for disabled individuals |
| Emo Wallpapers | IOS | 0 | 3.6 | 16 | E | No active component | Providing quotes |
| eMoods Bipolar Mood Tracker | AND | 100000 | 4.6 | 4085 | I | - |  |
| Emotions & Feelings Chart | IOS | 0 | 5 | 16 | E | Focus not depression | Focus on emotions but not for depression |
| engage warfarin self-care | NHS |  |  |  | E | Focus not depression | Focus on self-care not mental health |
| Enlighten | IOS | 0 | 4.9 | 34 | I | - |  |
| eQuoo: Emotional Fitness Game | NHS |  |  |  | E | Focus not depression | Game for emotional fitnes |
| Essential Emotions | IOS | 0 | 4.6 | 15 | E | Focus not depression | Focus on emotions but not for depression |
| Evergreen Life | NHS |  |  |  | E | Focus not depression | Focus on massage not depression |
| Everyday Health with Acupressure - Daily Massage! | IOS | 0 | 2 | 1 | E | No access | No access |
| Exercise Depression | IOS/AND | 100 | NA | NA | I | - |  |
| EXi | NHS |  |  |  | E | Focus not depression | Physical Activity App |
| Fabulous: Daily Planner & Self Care | AND | 5000000 | 4.5 | 307819 | E | Focus not depression | Motivation and habit tracker |
| Family Assist | NHS |  |  |  | E | Focus not depression | Focus on family assistance |
| Fax App | APA | 5000000 | 4.5 | 307819 | E | Focus not depression | APA professional recommendation to fax PDFs |
| Fear Tools - Anxiety Aid | AND | 50000 | 4.3 | 107 | E | Focus not depression | Focus on anxiety and not depression |
| Feedly | APA |  |  |  | E | Focus not depression | Organizing news feed of news outlets, journals, blogs and YouTube Channels |
| feel better - Mood & CBT therapy to manifest goals | AND | 50000 | 4.3 | 821 | I | - |  |
| ‎Feeling Good: positive mindset | AND | 0 | 4.1 | 46 | D | D |  |
| Feeling Good: positive mindset | NHS | 0 | 4.1 | 46 | I | - | NHS services |
| Feely - Take control of your mental health and feel happier | IOS | 0 | 1.3 | 6 | I | - |  |
| Fidget Spinner Feel | IOS | 0 | 4.1 | 85 | E | Focus not depression | Game for stress relieve |
| Fight depression | AND | 1000 | 4.9 | 7 | E | No active component | Religious quotes and images |
| Fight Depression - Take Baby Steps to Conquer It | AND | 50 | NA | NA | E | No active component | Providing quotes |
| Fight Depression Naturally - Educationfirm | AND | 5 | NA | NA | E | No access | No access |
| Fight Depression Naturally - Tipsbook | IOS | 50000 | 4.4 | 370 | I | - |  |
| Fight Depression Naturally Guide | AND | 100 | NA | NA | I | - |  |
| Fight Depression Naturally PRO | AND | 100 | 4.5 | 6 | I | - |  |
| First Aid by British Red Cross | NHS |  |  |  | E | Focus not depression | App of the British Red Cross |
| Flat Tomato | APA | 100 | 4.5 | 6 | E | Focus not depression | APA professional recommendation to focus using timer |
| Flow depression | AND | 1000 | 4.8 | 5 | I | - |  |
| Fox 26 Houston Weather – Radar | IOS | 0 | 3.9 | 48 | E | Focus not depression | Focus on meditation without depression focus |
| Free Guided Meditation, Sleep and Relaxation | AND | 10000 | 4 | 52 | E | Focus not depression | Focus on meditation without depression focus |
| Friend Shoulder: Advices and Vent Anonymous - chat | AND | 1000000 | 4.3 | 30240 | I | - |  |
| GDm-Health | NHS |  |  |  | E | Focus not depression |  |
| Geriatric Depression Scale | IOS | 0 | NA | NA | E | No active component | Depression triage without any intervention components |
| Geriatric Depression Scale 15 | IOS | 0 | 3.3 | 10 | E | No active component | Depression triage without any intervention components |
| Geriatric Depression Scale 2.0 | AND | 1000 | 5 | 1 | E | No active component | Depression triage without any intervention components |
| Get relaxed free! - Personal Hypnosis Program | IOS | 0 | 1 | 1 | E | Focus not depression | Focus on hypnosis without depression focus |
| Get Rid Of Depression with Acupressure Massage! | IOS | 0 | 1 | 1 | E | Focus not depression | Focus on massage not depression |
| GG Confidence & Self Esteem | IOS | 0 | 4.7 | 129 | E | Focus not depression | Focus on self-esteem not depression |
| GG Self Care CBT Daily Journey | IOS | 0 | 4.7 | 53 | E | Focus not depression | Focus on stress, anxiety and mood but not depression |
| Ginger Emotional Support | IOS | 0 | 4.4 | 328 | E | Focus not depression | Focus on " reduce stress and anxiety, strengthen your relationships, increase productivity" not depression |
| Gong Bath - Gong Sounds Relax | IOS | 0 | 5 | 1 | E | Focus not depression | Presenting a gong sound |
| Good News Network | IOS | 0 | 4.6 | 233 | E | Focus not depression | Reporting good news |
| Goodreads | APA |  |  |  | E | Focus not depression | APA self-care recommendation for good books |
| Google Analytics | APA |  |  |  | E | Focus not depression | APA professional recommendation to analyze trends |
| Google Keep | APA | 0 | 4.6 | 233 | E | Focus not depression | APA professional recommendation to store notes |
| GrowApp - Self-Care Assistant | IOS | 0 | 4.7 | 165 | E | No access | No access |
| Guide Meditation | IOS | 0 | 5 | 22 | E | Focus not depression | Focus on meditation without depression focus |
| Guided Meditation for sleep | IOS | 0 | 4 | 7 | E | Focus not depression | Focus on meditation without depression focus |
| Guided Mind | IOS | 0 | 4.7 | 319 | E | Focus not depression | Focus on meditation without depression focus |
| Guided Sleep Meditation - Relieve Insomnia Helper | IOS | 0 | 4.8 | 128 | E | Focus not depression | Focus on meditation without depression focus |
| Hamilton Depression Rating Scale (HAMD-7) | IOS | 0 | NA | NA | E | No active component | Depression triage without any intervention components |
| Happier You - Community, therapy | IOS | 0 | 4.7 | 76 | E | No active component | Community platform to interact without intervention components |
| Happify | ADAA |  |  |  | D | D | D |
| Happify: for Stress & Worry | IOS/AND | 500000 | 4.5 | 2479 | I | - |  |
| Happy - A Mental Health App | IOS | 0 | 5 | 17 | I | - |  |
| Happy Being | IOS | 0 | 5 | 1 | E | Focus not depression | Focus on happiness and not depression |
| Happy Boost, Depression Help | IOS | 0 | 4.8 | 58 | I | - |  |
| Headspace | ADAA | 0 | 4.8 | 58 | I | - | Diabetes, Pregnancy and baby |
| Heal Yourself NOW: Mindful Meditations for Healing | IOS | 0 | 4.5 | 4 | E | Focus not depression | Focus on meditation without depression focus |
| Healthcare Anywhere | NHS |  |  |  | E | Focus not depression | Diabetes, Pregnancy and baby |
| Healthera | NHS |  |  |  | E | Focus not depression | Diabetes, Pregnancy and baby |
| Healthpiper -Psychiatrist Chat | IOS | 0 | 4.7 | 46 | E | Focus not depression | Chat with a psychiatrist - not an intervention |
| HealthUnlocked | NHS |  |  |  | E | Focus not depression | Diabetes, Pregnancy and baby |
| HealthUnlocked Communities | IOS | 0 | 4.9 | 1012 | E | Focus not depression | Community platform to interact without intervention components |
| HearMe.app | IOS | 0 | 4.8 | 129 | I | - |  |
| HelloMind - Hypnosis & Therapy | IOS | 0 | 4 | 118 | E | Focus not depression | Focus on meditation without depression focus |
| HERE - Meditation | IOS | 0 | 4.8 | 106 | E | Focus not depression | Focus on meditation without depression focus |
| Hidden Feeling Quotes - Heart Touching Quotes | AND | 100000 | 4.7 | 984 | E | Focus not depression | Focus on emotions but not for depression |
| hiMoment - Gratitude Journal | IOS | 0 | 3.8 | 19 | E | Focus not depression | Focus on gratitude |
| HOLD - Stress Help & Self-Care | IOS | 0 | 5 | 6 | E | Focus not depression | Focus on stress-relief not depression |
| Home Remedies For Depression | AND | 100 | NA | NA | E | No access | No access |
| Hospify | NHS |  |  |  | E | Focus not depression | Diabetes, Pregnancy and baby |
| How to Be Mentally Strong Tips | AND | 10000 | 4.5 | 102 | E | Focus not depression | Focus on mental health not depression |
| How To Beat Depression | AND | 500 | NA | NA | I | - |  |
| How To Cure Depression | AND | 50 | NA | NA | I | - |  |
| How To Deal With Depression - Amamiya Apps | AND | 10 | NA | NA | E | No active component | Information about depression |
| How to Deal With Depression - ARUNAS APPS LLP | IOS/AND | 100 | NA | NA | E | No active component | Information about depression |
| How To Deal With Depression - FluffyCuteApps | AND | 100 | NA | NA | I | - |  |
| How to deal with depression - Koodalappz | IOS/AND | 100 | NA | NA | D | D |  |
| How to deal with depression - Koodalappz | AND | 100 | NA | NA | I | - |  |
| How to Get Over Depression | AND | 1000 | 2.5 | 2 | I | - |  |
| How To Overcome Depression - Bestappsforyou | AND | 1000 | 3 | 6 | I | - |  |
| How To Overcome Depression - FluffyCuteApps | AND | 1000 | 3.6 | 8 | I | - |  |
| How To Overcome Depression, Deal With Depression | AND | 100 | NA | NA | E | No active component | Information about depression |
| Howz | NHS |  |  |  | E | Focus not depression | Online community, Body areas |
| Hub of Hope | IOS | 0 | 4.9 | 15 | E | Focus not depression | Focus on hope not depression |
| Humm.ly - Live Better w/ Music | IOS | 0 | 4.8 | 1327 | E | Focus not depression | Focus on music and improved living |
| Hypnosis for Anxiety, Stress and Depression Guide | AND | 100 | NA | NA | I | - |  |
| I am - Positive Affirmations | IOS | 0 | 4.8 | 20037 | E | Focus not depression | Focus on positive affirmations |
| I meet Myself | AND | 100000 | 4 | 1910 | E | Focus not depression | Game for self-discovery |
| iCBT App | ADAA |  |  |  | E | Focus not depression | Online community, Body areas |
| iCouch CBT | IOS | 0 | 5 | 1 | I | - |  |
| ieso | IOS | 0 | 0 | 0 | D | D |  |
| ieso | NHS | 0 | 0 | 0 | E | No access | NHS services |
| ifeel: Modern Online Therapy | IOS | 0 | 4.8 | 26 | I | - |  |
| iFORA O2 | IOS | 0 | 4.1 | 14 | E | Focus not depression | Sleep tracking |
| iMood Journal - Mood Diary | IOS | 0 | 4.4 | 132 | E | Focus not depression | Mood tracking |
| In Hand - A tool to focus where you're at and bring back the balance | IOS | 0 | 2.3 | 3 | E | Focus not depression | Mood tracking |
| InnerHour Self-Care Therapy - Anxiety & Depression | IOS/AND | 500000 | 4.3 | 4643 | I | - |  |
| Inspirational Happiness Tips! | IOS | 0 | 4.6 | 52 | E | Focus not depression | Providing quotes |
| Inspirational Quotes For Depression | AND | 10 | NA | NA | E | No active component | Providing quotes |
| Inspire | IOS | 0 | 4.8 | 342 | E | Focus not depression | Information about general health from patients and caregivers |
| Integrated Family Delivered Neonatal Care (IFDC) | NHS |  |  |  | E | Focus not depression | NHS services |
| Intellicare | APA | 0 | 4.8 | 342 | E | Focus not depression | Platform for a number of different apps targeting mental health |
| IntelliCare Hub | IOS/AND | 500 | 5 | 5 | E | Focus not depression | Recommendation app to download a number of mental health apps |
| iPrevail: Anxiety & Depression | AND | 10000 | 2.9 | 132 | I | - |  |
| Jade - Mood Tracker, Diary, Journal | IOS/AND | 10000 | 4.5 | 318 | I | - |  |
| JOI: Self-Care Mood Journal | IOS | 0 | 4.3 | 16 | I | - |  |
| Jour: Journal for Mindfulness | IOS | 0 | 4.9 | 11334 | E | Focus not depression | Focus on mindfulness without depression focus |
| Journey: Live Wellness | IOS | 0 | 4.8 | 430 | E | No access | No access |
| Joyable: An AbleTo Program | IOS | 0 | 4.5 | 1351 | I | - |  |
| Kicks Count | NHS |  |  |  | E | Focus not depression | Healthy living, Covid |
| Kintsugi | IOS | 0 | 4.5 | 103 | E | Focus not depression | Focus on self-care not depression |
| Let's Meditate: Sleep & Guided Meditation | AND | 1000000 | 4.8 | 50271 | E | Focus not depression | Focus on meditation without depression focus |
| Life Hack Tips - Daily Tips for your Life | AND | 500000 | 4.7 | 25202 | E | Focus not depression | Daily life hacks not depression |
| Life Reboot - Fight Depression | AND | 1000 | 3.7 | 137 | I | - |  |
| Life Tracker & Healthy Living | IOS | 0 | 4.3 | 211 | E | Focus not depression | Focus on healthy living |
| Life: Personal Diary, Journal, Notebook | AND | 500000 | 4.5 | 24907 | E | Focus not depression | Focus on healthy living and personal development |
| Lifehelp - Online Therapy | IOS | 0 | 3.9 | 33 | I | - |  |
| Lift - Depression & Anxiety | AND | 1000 | 4.5 | 27 | I | - |  |
| Liv Happiness Companion | IOS | 0 | 4.5 | 624 | E | Focus not depression | Focus on happy living |
| Liva UK | NHS |  |  |  | E | Focus not depression | Mental health, NHS services |
| Live Happy | IOS | 0 | 4.5 | 48 | E | Focus not depression | Focus on disease management and lifestyle change |
| Live Life - Self Help Hypnosis | IOS | 0 | 4.8 | 12 | E | Focus not depression | Focus on hypnosis without depression focus |
| Living With | NHS |  |  |  | E | Focus not depression | Pregnancy and baby, Child health |
| Lonely wallpaper | IOS/AND | 100000 | 4.5 | 627 | E | Focus not depression | Providing loneliness quotes |
| Loner | AND | 1000000 | 4.6 | 28945 | E | Focus not depression | Focus on loneliness |
| Low Carb Program | NHS |  |  |  | E | Focus not depression |  |
| Lyf! You're not alone | IOS | 0 | 4.5 | 100 | E | Focus not depression | Focus on anxiety, infidelity, coping with loss, struggling with addiction, or questioning your sexual identity |
| Lyftly: Wellness & Happiness | IOS | 0 | 4.9 | 82 | E | Focus not depression | Focus on wellness |
| LymEx | NHS |  |  |  | E | Focus not depression | Pregnancy and baby, Child health |
| Mandala Coloring Book | IOS | 0 | 4 | 200 | E | Focus not depression | Game for stress relieve |
| mapmydiabetes | NHS |  |  |  | E | Focus not depression | Pregnancy and baby, Child health |
| Me v PMDD - Symptom Tracker | IOS | 0 | 2.7 | 6 | E | Focus not depression | Special focus on PMDD |
| Meditation & Relaxation Music: Calm Sleep Sounds | AND | 1000000 | 4.6 | 28280 | E | Focus not depression | Audio for meditation without depression |
| MEDITATION BY MT - SELF HELP, STRESS RELIEF | IOS/AND | 10000 | 4.4 | 90 | E | Focus not depression | Focus on meditation without depression focus |
| Meditation For Sleep - Refresh | IOS | 0 | 4.8 | 907 | E | Focus not depression | Focus on meditation without depression focus |
| Medloop | NHS |  |  |  | E | Focus not depression | Pregnancy and baby |
| Meetup | APA | 10000000 | 4.2 | 99168 | D | D |  |
| Meetup | IOS/AND | 10000000 | 4.2 | 99168 | E | Focus not depression | Find events online |
| MeeTwo | NHS |  |  |  | E | Focus not depression |  |
| MellowMe:Meditation & Breathe | IOS | 0 | 4.7 | 866 | E | Focus not depression | Focus on meditation without depression focus |
| Mend: Breakup & Divorce Guide | IOS | 0 | 4.1 | 660 | E | Focus not depression | Focus on breakup and divorce |
| Mental health tests | IOS/AND | 100000 | 4 | 859 | E | Focus not depression | Tests for mental health in general |
| Merrier - Anxiety Relief Games | IOS | 0 | 5 | 24 | E | Focus not depression | Game for anxiety |
| Miiskin | NHS |  |  |  | E | Focus not depression | Diabetes, Healthy living |
| Mindful Moon - Stress Relief & Happiness through Inspirational Quotes & Positive Daily Reminders | IOS | 0 | 4 | 14 | E | Focus not depression | Focus on meditation without depression focus |
| Mindfulness coach | AND | 100000 | 4.7 | 3802 | I | - |  |
| Mindfulness For Depression | IOS/AND | 5000 | 4 | 13 | I | - |  |
| Mindfulness: Finding peace | IOS | 0 | 4.5 | 273 | E | Focus not depression | Focus on mindfulness without depression focus |
| Mindset: Hypnotherapy & Sleep | IOS | 0 | 4.4 | 331 | E | Focus not depression | Focus on hypnosis without depression focus |
| MindShift | ADAA |  |  |  | E | Focus not depression | Diabetes, Healthy living |
| MindShift CBT - Anxiety Canada | IOS/AND | 100000 | 4.2 | 1190 | E | Focus not depression | Focus on anxiety and not depression |
| Mindventure: Daily Self Care | IOS | 0 | 4.8 | 10 | E | Focus not depression | Focus on self-care not depression |
| MoleCare | NHS |  |  |  | E | Focus not depression | Diabetes |
| Mood & Anxiety Diary | IOS | 0 | 3.8 | 4 | E | Focus not depression | Mood and anxiety not depression |
| Mood App: Journal | IOS | 0 | 4.6 | 3216 | E | Focus not depression | Mood tracking |
| Mood Balance - Daily Tracker | IOS | 0 | 4.5 | 415 | E | Focus not depression | Mood tracking |
| Mood log | AND | 50000 | 4.4 | 1169 | I | - |  |
| Mood Mint - Boost Your Mood | IOS | 0 | 4.7 | 53 | E | Focus not depression | Mood tracking |
| Mood Patterns - a mood tracker with privacy | AND | 10000 | 4.5 | 313 | E | Focus not depression | Mood tracking |
| Mood Ring: Your Emoji Journal | IOS | 0 | 4.5 | 42 | E | Focus not depression | Mood tracking |
| Mood Tracker - Bipolar Mood Journal, Mood Diary | IOS/AND | 1000 | 3.2 | 18 | I | - |  |
| Mood Tracker, Journal, Anti Depression Diary | AND | 500000 | 4.2 | 7128 | I | - |  |
| Mood Tracker, Journal, Diary - Anti Depression app | AND | 5000 | 4.3 | 7497 | I | - |  |
| Mood-Diary | IOS | 0 | 1 | 1 | I | - |  |
| Moodboost | IOS | 0 | 4.1 | 47 | E | Focus not depression | Mood tracking |
| MoodDiary BETA | AND | 10000 | 4.3 | 602 | I | - |  |
| Moodfit - Fitness for your mental health | IOS/AND | 10000 | 4.5 | 360 | I | - |  |
| Moodflow: Mood Tracker, Year in Pixels, Diary | AND | 50000 | 4.7 | 1129 | E | Focus not depression | Mood tracking |
| Moodily - mood tracker, depression support | IOS/AND | 10000 | 3.3 | 34 | I | - |  |
| Moodistory Mood Tracker, Diary | IOS | 0 | 4.9 | 164 | I | - |  |
| Mooditude - Mood Tracker \| CBT | IOS | 0 | 4.7 | 6 | I | - |  |
| MoodKit | AND | 0 | 4.5 | 153 | D | D |  |
| MoodKit | ADAA/APA | 0 | 4.5 | 153 | I | - |  |
| MoodLinks - Anxiety & Depression | AND | 100 | 4.5 | 6 | I | - |  |
| MoodMission - Cope with Stress, Moods & Anxiety | AND | 10000 | 3.3 | 181 | I | - |  |
| Moodnotes - CBT & Mood Tracker | IOS | 0 | 4.6 | 7639 | I | - |  |
| MoodPanda | IOS | 0 | 4.1 | 8 | I | - |  |
| Moodpath - Depression & Anxiety Test | IOS/AND | 1000000 | 4.6 | 27797 | I | - |  |
| Moods: Mental Health Tracking | IOS | 0 | 3.5 | 72 | E | Focus not depression | Mood tracking and training |
| MoodSpace - Stress, anxiety, & low mood self-help | AND | 100000 | 4.7 | 1784 | I | - |  |
| MoodTools | ADAA | 100000 | 4.3 | 3167 | I | - |  |
| MoodTools - Depression Aid | AND | 100000 | 4.4 | 3093 | D | D |  |
| Moodtrack Private Diary | IOS | 0 | 4.5 | 111 | E | Focus not depression | Mood tracking |
| Moodtrack Social Diary | IOS | 0 | 4.5 | 753 | E | Focus not depression | Mood tracking |
| MoodWell: mood journal tracker | IOS | 0 | 4.7 | 122 | E | Focus not depression | Mood tracking |
| Moody: Mood Tracker & Journal | IOS | 0 | 4.5 | 270 | E | Focus not depression | Mood tracking |
| Morning Pages – Daily Journal | IOS | 0 | 4.7 | 283 | E | Focus not depression | Mood tracking |
| Motivation - Daily quotes | AND | 100000 | 4.8 | 5422 | E | Focus not depression | Providing quotes |
| Move On with AJ | IOS | 0 | 5 | 5 | E | Focus not depression | Lifiting spirit in times of trouble |
| Mumoactive | NHS |  |  |  | E | Focus not depression | Cancer |
| Music Therapy for depression | AND | 1000 | 3.5 | 23 | I | - |  |
| MUTU System Programme | NHS |  |  |  | E | Focus not depression | Cancer |
| My Diabetes My Way | NHS |  |  |  | E | Focus not depression | Cancer |
| My Health Guide | NHS |  |  |  | E | Focus not depression | Cancer |
| My House of Memories | NHS |  |  |  | E | Focus not depression | Cancer |
| My Mantra | AND | 100 | 5 | 2 | E | Focus not depression | Developing mantras and simple phrases and images to remember positive things in your daily life |
| my mhealth: myAsthma | NHS |  |  |  | E | Focus not depression | Cancer |
| my mhealth: myCOPD | NHS |  |  |  | E | Focus not depression | Cancer |
| my mhealth: myDiabetes | NHS |  |  |  | E | Focus not depression | Cancer |
| my mhealth: myHeart | NHS |  |  |  | E | Focus not depression | Cancer |
| ‎My Online Therapy: Counselling | IOS | 0 | 4.1 | 112 | E | No active component | Tele-therapy video-consultation |
| My Possible Self: The Mental Health App | NHS |  |  |  | E | Focus not depression | Cancer |
| MyChoicePad | NHS |  |  |  | E | Focus not depression | Cancer |
| MyCognition Home | NHS |  |  |  | E | Focus not depression | Cancer |
| MyDesmond | NHS |  |  |  | E | Focus not depression | Cancer |
| myGP | NHS |  |  |  | E | Focus not depression | Cancer |
| MyMoodTracker | IOS | 0 | 4.3 | 13 | I | - |  |
| MyPossibleSelf: Mental Health | IOS | 0 | 3.5 | 44 | I | - |  |
| myStrength | AND | 10000 | 4.5 | 189 | I | - |  |
| mySugr | NHS |  |  |  | E | Focus not depression | Cancer |
| Natural Depression Treatment | AND | 100 | NA | NA | I | - |  |
| Neuronation Ease: Mindfulness | IOS | 0 | 4.3 | 11 | E | Focus not depression | Focus on mindfulness without depression focus |
| NeuroX Depression | AND | 1000 | NA | NA | I | - |  |
| NeuroX Depression PRO | IOS | 0 | NA | NA | I | - |  |
| New! Depression Test | IOS | 0 | 4 | 13 | E | No active component | Depression triage without any intervention components |
| NHS App | NHS |  |  |  | E | Focus not depression | Learning disabilities, NHS services, Social care |
| NHS COVID-19 | NHS |  |  |  | E | Focus not depression | Memory and communication, Social care |
| NHS Go | NHS |  |  |  | E | Focus not depression | Memory and communication, Social care |
| NHS Online Bexley | NHS |  |  |  | E | Focus not depression | Respiratory |
| NHS Pulse | NHS |  |  |  | E | Focus not depression | Respiratory |
| notOK | IOS | 0 | 4.2 | 42 | E | Focus not depression | Panic Button for friends and familiy |
| nujjer | NHS |  |  |  | E | Focus not depression | Body areas |
| O2 Care - SpO2 HRV Biofeedback | IOS | 0 | 2.6 | 16 | E | Focus not depression | Breathing training |
| OCD Daily Exercise by GG (GGOC) | AND | 10000 | 4.5 | 183 | E | Focus not depression | Focus on OCD and anxiety |
| OCD Test Y-BOCS | IOS | 0 | 5 | 14 | E | Focus not depression | Focus on OCD and anxiety |
| OneTouch Reveal | NHS |  |  |  | E | Focus not depression | Memory and communication |
| Online Counseling - Treat Depression and Anxiety | AND | 1000 | 4.7 | 17 | I | - |  |
| Online therapy - mental help. Support groups. | AND | 500000 | 4.4 | 4654 | I | - |  |
| Overcome depression | AND | 10 | NA | NA | I | - |  |
| Overcome Depression - How to Cope with Depression | AND | 50 | NA | NA | I | - |  |
| Overcome Depression and Anxiety | AND | 500 | NA | NA | I | - |  |
| Overcoming depression - Eriksson Softech | AND | 1000 | 4.5 | 2 | E | No active component | Information about depression |
| Oviva | NHS |  |  |  | E | Focus not depression | Diabetes |
| OWise breast cancer | NHS |  |  |  | E | Focus not depression | Diabetes |
| Pando | NHS |  |  |  | E | Focus not depression | Diabetes |
| Panic Relief | ADAA |  |  |  | E | Focus not depression | Diabetes |
| Pathway through Pain | NHS |  |  |  | E | Focus not depression | NHS services |
| Patients Know Best | NHS |  |  |  | E | Focus not depression | Covid, NHS services |
| PatientsLikeMe | IOS | 0 | 4.6 | 1175 | E | No active component | Patients community platform |
| Patronus: Mental Health Guide | IOS | 0 | 4.8 | 211 | E | Focus not depression | Focus on emotional wellbeing not depression |
| PE Coach | ADAA |  |  |  | E | Focus not depression | NHS services, Covid |
| Peace: Calm, Sleep, Meditation | AND | 50000 | 4.1 | 176 | I | - |  |
| Peanut | NHS |  |  |  | E | Focus not depression | Diabetes, Healthy living |
| Peptalk Motivation | IOS | 0 | 4.8 | 5946 | E | Focus not depression | Providing motivation |
| Personality Test (Psychology): Rorschach Test | AND | 100000 | 4.1 | 4156 | E | Focus not depression | Personality test and not depression |
| PerSoNClinic ( Depression, Chronic Pain, Cancer) | AND | 1000 | 4.6 | 9 | I | - |  |
| PHQ-9 Depression Test Questionnaire | IOS | 0 | 3.2 | 5 | E | No active component | Depression triage without any intervention components |
| Pin it or Bin it | IOS | 0 | 4.7 | 13 | E | Focus not depression | CBT exercise without a focus on depression |
| PinkyMind - Online counseling & therapy chat app | AND | 10000 | 4.6 | 305 | I | - |  |
| Pluck It: hairs and emotions | AND | 100000 | 4.8 | 22382 | E | Focus not depression | Game for plucking hair |
| Pocket Pharmacist | APA | 0 | 4.7 | 1700 | E | Focus not depression | Information about different drugs |
| Pointo: Talk therapy, mood journal, self-reflection | IOS/AND | 10000 | 4.7 | 282 | I | - |  |
| Postpartum depression - Pinkdev | AND | 500 | NA | NA | E | Focus not depression | Focus on postpartum depression |
| Prana Breath: Calm & Meditate | AND | 1000000 | 4.7 | 59128 | E | Focus not depression | Focus on meditation without depression focus |
| Pride Counseling | IOS | 0 | 3.8 | 683 | E | Focus not depression | Online counselling for LGBTQ |
| PsychApp – Depression, Anxiety, Panic Attack (PRO) | AND | 100 | 5 | 9 | I | - |  |
| PsychApp free | AND | 5000 | 3.8 | 12 | I | - |  |
| PsychExplorer | APA |  |  |  | E | Focus not depression | Showing videos related to psychology |
| Psychology Book - 1000+ Amazing Psychology Facts | AND | 100000 | 4.6 | 5832 | E | Focus not depression | Information about depression |
| Psychology Chat - Help in Psychologist role online | AND | 10000 | 4.4 | 536 | I | - |  |
| Psychosomat Depression Burnout | AND | 10000 | 4.4 | 117 | I | - |  |
| Psychotherapy Networker | IOS | 0 | 4.8 | 308 | E | Aimed At Professionals | Magazine for mental health professionals |
| PsyTests | AND | 1000000 | 4.6 | 81014 | E | No active component | Depression triage without any intervention components |
| PTSD Coach | ADAA |  |  |  | E | Focus not depression | Pregnancy and baby, Online community |
| Punkt: One-sentence journal | IOS | 0 | 4.8 | 18 | E | Focus not depression | Journaling with no focus on depression |
| Pzizz | NHS |  |  |  | E | Focus not depression | Pregnancy and baby, Online community |
| Quality Mind Global | IOS | 0 | 5 | 10 | E | Focus not depression | Thought shopping, meditation, "step by step program information as you progress" |
| Quirk CBT | IOS | 0 | 4.3 | 33 | E | Focus not depression | CBT thought diary app without focus on depression |
| Quote Depression Wallpapers HD | AND | 10 | NA | NA | E | No active component | Providing quotes |
| Rafi-Tone | NHS |  |  |  | E | Focus not depression | Pregnancy and baby, Online community |
| RareGuru: Rare Diseases | IOS | 0 | 5 | 43 | E | Focus not depression | Focus on rare diseases not depression |
| Recovery Path: Addiction Recovery & Addiction Help | AND | 10000 | 4.6 | 973 | E | Focus not depression | Focus on addiction |
| Reduce depression | AND | 1000 | 4.1 | 8 | I | - |  |
| Reflectly - Journal / Diary | IOS/AND | 1000000 | 4.3 | 28228 | E | Focus not depression | Journaling with no focus on depression |
| Reiki Relaxation Hypnosis | IOS | 0 | 4.7 | 69 | E | Focus not depression | Focus on hypnosis without depression focus |
| Relationship Advice & Help: Emotional Support Free | AND | 100000 | 4.6 | 2309 | E | Focus not depression | Focus on relationship advice |
| Relax & Sleep Well: Hypnosis and Meditation | IOS/AND | 500000 | 4.7 | 6968 | E | Focus not depression | Focus on hypnosis without depression focus |
| Relax Melodies | APA |  |  |  | E | Focus not depression | Sounds for relaxation |
| Relaxia ~ Sleep aid, Relaxation & Yoga Meditation with Ambient Sound-scapes inspired by Nature | IOS | 0 | 4.5 | 121 | D | D |  |
| Relaxia Free: Sleep aid, Relaxation, Meditation Yoga, Ambient Soundscapes inspired by Nature | IOS | 0 | 4.2 | 37 | E | Focus not depression | Focus on meditation without depression focus |
| ReliefLink | APA | 0 | 3.9 | 7 | E | Focus not depression | Suicide prevention |
| Relieve Depression Hypnosis - Mood & Anxiety Help | AND | 50000 | 4.6 | 380 | I | - |  |
| Remente: Self Improvement | AND | 500000 | 4.3 | 9717 | I | - |  |
| Replica: My AI Friend | IOS/AND | 1000000 | 4.6 | 165950 | E | Focus not depression | Chatbot for interaction without a depression focus |
| Reservoire - Build Resilience | IOS | 0 | 5 | 202 | E | No active component | Network to build personal resilience and increase health intelligence |
| rTribe - Coaching for Anxiety / Addiction / Depression | AND | 100000 | 4.3 | 3710 | I | - |  |
| Sad and Depressed Wallpapers | AND | 1000 | 4.3 | 7 | E | No active component | Providing quotes |
| Sad And Depression Quotes 2020 | AND | 10 | NA | NA | E | No active component | Providing quotes |
| Sad Lonely Painful & Hurt Love | AND | 100000 | 4.5 | 2553 | E | No active component | Providing quotes |
| Sad Quote Wallpapers - Modux Apps | AND | 10000 | 4.3 | 61 | E | No active component | Providing quotes |
| Sad Quote Wallpapers - Leafgreen | AND | 500000 | 4.4 | 2227 | E | No active component | Providing quotes |
| Sad quotes | AND | 10000 | 4.2 | 44 | E | No active component | Providing quotes |
| Sad wallpaper HD | IOS/AND | 100000 | 4.3 | 610 | E | No active component | Providing quotes |
| Sad wallpaper: Unhappy quote, anime sad wallpaper | AND | 100000 | 4.4 | 5452 | E | No active component | Providing quotes |
| Sad Wallpapers - Atlas Labs | AND | 0 | 4.6 | 40 | E | No active component | Providing quotes |
| Sad Wallpapers - wallpaper studio pro | AND | 10000 | 4 | 47 | E | No active component | Providing quotes |
| Sad Wallpapers - Droid Wallpaperss | AND | 10000 | 4.2 | 31 | E | No active component | Providing quotes |
| Sad Wallpapers - Modux Apps | AND | 50000 | 4.4 | 134 | E | No active component | Providing quotes |
| Sad Wallpapers - soko Apps | IOS | 500000 | 4.5 | 3223 | E | No active component | Providing quotes |
| Sad Wallpapers HD Backgrounds 2020 | AND | 5000 | 3.7 | 15 | E | No active component | Providing quotes |
| Sad Wallpapers Lockscreen | AND | 10000 | 4.5 | 39 | E | No active component | Providing quotes |
| Sanvello for Stress, Anxiety & Depression | IOS/AND | 1000000 | 4.7 | 15771 | I | - |  |
| ‎Sayana: Emotional Self-Care on the App Store | IOS | 0 | 4.7 | 6400 | E | Focus not depression | Focus on self-care not depression |
| Scouse Guru | IOS | 0 | 5 | 22 | E | Focus not depression | Focus on meditation without depression focus |
| Second Nature | NHS |  |  |  | E | Focus not depression | Respiratory, Child health |
| Self Help | IOS | 0 | 4.7 | 37 | E | Focus not depression | Self-help libary by NHS |
| Self-Care & Vent : Helponymous | IOS | 0 | 4.9 | 102 | E | Not English | Not English |
| Self-Esteem Hypnosis - Positive Daily Affirmations | AND | 50000 | 4.3 | 347 | E | Focus not depression | Affirmations and self-esteem hypnosis |
| Self-Help Anxiety Management | ADAA |  |  |  | E | Focus not depression | Respiratory, Child health |
| Self-manage depression: Daily exercise (GGDE) | AND | 1000 | 4.2 | 41 | I | - |  |
| Serenity: Guided Meditation & Mindfulness | IOS/AND | 100000 | 4.8 | 11631 | E | Focus not depression | Focus on meditation without depression focus |
| Serenity: Guided Mental Health | AND | 5000 | 3.9 | 80 | I | - |  |
| Serenity: Meditation | IOS | 0 | 4.8 | 312 | E | Focus not depression | Focus on meditation without depression focus |
| Sideline | APA |  |  |  | E | Focus not depression | Focus on meditation without depression focus |
| Signs & Symptoms Depression | IOS | 0 | NA | NA | E | No active component | Information about depression |
| SilverCloud | AND | 1000 | 3.1 | 53 | D | D |  |
| SilverCloud | NHS | 1000 | 3.1 | 53 | E | No access | Respiratory, Child health |
| Simple DBT Skills Diary Card | IOS | 0 | 4.3 | 84 | E | Focus not depression | Focus on DBT not Depression |
| Simple Depression Test | IOS | 0 | 5 | 3 | E | No active component | Depression triage without any intervention components |
| Simple Practice | APA |  |  |  | E | Focus not depression | APA professional recommendation to scheduling clients, billing and claims |
| SingFit | IOS | 0 | 4.4 | 7 | E | Focus not depression | Singing coach |
| Sleep & meditation; sleep stories to relax by Wysa | AND | 10000 | 4.7 | 742 | E | Focus not depression | Focus on meditation without depression focus |
| Sleep School for Professionals | IOS | 0 | 3.7 | 9 | E | Focus not depression | Focus on sleep not depression |
| Sleep Tracker + Lifestyle | IOS | 0 | 4.2 | 26 | E | Focus not depression | Focus on sleep not depression |
| Sleep Well Hypnosis - For Insomnia & Deep Sleep | IOS/AND | 100000 | 4.2 | 2208 | I | - |  |
| Sleepio | NHS |  |  |  | E | Focus not depression | Respiratory, Child health |
| Sleepstation | NHS | 100000 | 4.2 | 2208 | E | Focus not depression | Diabetes, Healthy living |
| Smash It! AR - Stress Relief | IOS | 0 | 4.6 | 64 | E | Focus not depression | Focus on stress-relief not depression |
| Smilers - helping with depression in Arabic | IOS | 0 | NA | NA | E | Not English | Not English |
| SoberTool - Addiction Recovery | IOS | 0 | 4.7 | 2312 | E | Focus not depression | Focus on addiction |
| Sooma Depression Therapy Pro | AND | 1000 | 4 | 6 | I | - |  |
| SOS QR | NHS |  |  |  | E | Focus not depression | Diabetes, Healthy living |
| Soulspace Christian Meditation | IOS | 0 | 4.9 | 188 | E | Focus not depression | Christian quotes |
| Sparkle: Self-Care Checklist, Tracker & Journal | AND | 10000 | 4.6 | 72 | E | Focus not depression | Self-care focus |
| SpineWise | NHS |  |  |  | E | Focus not depression | Diabetes, Healthy living |
| Squeezy | NHS |  |  |  | E | Focus not depression | Diabetes, Healthy living |
| Squeezy CF | NHS |  |  |  | E | Focus not depression | Diabetes, Healthy living |
| Squeezy for men | NHS |  |  |  | E | Focus not depression | Mental health |
| Step Away | APA |  |  |  | E | Focus not depression | Focus on alcohol addiction |
| Stoa: Stoic Meditation & Diary | IOS | 0 | 4.6 | 158 | E | Focus not depression | Focus on meditation without depression focus |
| STOP Depression | IOS/AND | 10000 | 3.5 | 76 | E | No active component | Depression triage without any intervention components |
| Stop Panic & Anxiety Self-Help | IOS/AND | 100000 | 4.6 | 2374 | E | Focus not depression | Focus on anxiety and panic disorder |
| STOPP app | IOS | 0 | 3.6 | 5 | E | No active component | Providing quotes |
| Strategic Breathing | IOS | 0 | 4.9 | 175 | E | Focus not depression | Breathing training |
| Stress & Anxiety Companion | NHS |  |  |  | E | Focus not depression | Focus on Stress and Anxiety |
| Stress Anxiety Relief | IOS | 0 | 4.5 | 38 | E | Focus not depression | Focus on Stress and Anxiety |
| Stress Anxiety Relief Games | IOS | 0 | 4.6 | 553 | E | Focus not depression | Focus on Stress and Anxiety |
| Stress Control Norbu - meditation, relaxing games | AND | 10000 | 4.4 | 111 | E | Focus not depression | Focus on Stress and Anxiety |
| Stress Relief & Management App | IOS | 0 | 4.4 | 127 | E | Focus not depression | Focus on Stress and Anxiety |
| Stress Relief Relax Meditation | IOS | 0 | 4.5 | 90 | E | Focus not depression | Focus on Stress and Anxiety |
| StressScan - check your stress | IOS | 0 | 4 | 163 | E | Focus not depression | Focus on Stress and Anxiety |
| StressScan: heart rate monitoring and stress test | IOS/AND | 500000 | 3.9 | 2845 | E | Focus not depression | Focus on Stress and Anxiety |
| Student Health App | NHS |  |  |  | E | Focus not depression | Focus on students sleep |
| Sugarmedown | NHS | 500000 | 3.9 | 2845 | E | Focus not depression |  |
| Suicide Safety Plan | IOS | 0 | 4.9 | 17 | E | Focus not depression | Focus on suicide not depression |
| Sunny Sea Ocean Sleep Sounds | IOS | 0 | 4.8 | 282 | E | Focus not depression | Sleeping sounds |
| SuperBetter | IOS/AND | 100000 | 4.4 | 5965 | I | - |  |
| Supportive Amino | IOS | 0 | 4.9 | 27 | E | Focus not depression | Network for meeting new friends |
| Symptom Tracker: Pain History | IOS | 0 | 4.1 | 697 | E | Focus not depression | Pain tracking not depression |
| T2 Mood Tracker | AND | 100000 | 3.4 | 1847 | D | D |  |
| T2 Mood Tracker | ADAA | 100000 | 3.4 | 1847 | I | - | Body areas |
| Talk Around It Home | NHS |  |  |  | E | Focus not depression | Body areas |
| Talk, Breathe, Think | APA | 100000 | 3.4 | 1847 | E | Focus not depression | Breathing training |
| TalkCampus | AND | 1000 | 4.3 | 43 | I | - |  |
| TalkLife - Lonely, Stressed or Battling Anxiety? | IOS/AND | 500000 | 4.4 | 27896 | E | Focus not depression | Focus on loneliness |
| Talkspace Counseling & Therapy | AND | 100000 | 3 | 2339 | E | Focus not depression | Network for exchanging information and finding therapist |
| Tell A Buddy - Online Counseling & Life Management | IOS/AND | 5000 | 3.5 | 138 | E | Focus not depression | Network for exchanging information and finding therapist |
| The Anxiety Guy Audio Podcasts | IOS | 0 | 4.9 | 129 | E | Focus not depression | Focus on anxiety and not depression |
| The Breathing App | IOS | 0 | 4.3 | 29 | E | Focus not depression | Breathing training |
| The Depression Project | AND | 100 | 5 | 1 | E | No active component | Information from social media posts |
| The Firm | IOS | 0 | 3.9 | 59 | E | Focus not depression | Game |
| The Happy Child-Parenting App | IOS | 0 | 4.9 | 122 | E | Focus not depression | Parenting app |
| The Meaning of Life Experiment | IOS | 0 | 4.4 | 132 | E | Focus not depression | Focus on life quality not depression |
| The Mighty | IOS | 0 | 4.8 | 6895 | E | Focus not depression | Network for "mental health to chronic illness, disability, rare disease, parenting, neuro-diversity, care-giving" |
| The Szondi Test: Research of Depression | AND | 100000 | 4.1 | 3495 | E | No active component | Depression triage without any intervention components |
| The Tapping Solution | AND | 100000 | 4.7 | 2428 | E | Focus not depression | Focus on anxiety and stress |
| Therachat - journal and tracking | IOS | 0 | 4.1 | 75 | E | Focus not depression | Focus on mental fitness |
| Therapeer: Peer Emotional Support | AND | 10000 | 4.4 | 381 | I | - |  |
| TherapyChat - Online Therapy | IOS | 0 | 4.4 | 14 | E | Focus not depression | Tele-therapy video-consultation |
| Think-Ups | IOS | 0 | 4.6 | 26 | E | Focus not depression | Focus on Stress and Anxiety |
| Thinkladder | IOS | 0 | 3.3 | 15 | E | Focus not depression | Focus on "Perfectionism, Self-Worth, Social Distancing, Anxiety, Body Image, and Anger" |
| ThinkNinja | IOS | 0 | 3.8 | 29 | D | D |  |
| ThinkNinja | NHS | 0 | 3.8 | 29 | E | Focus not depression | Teens, parents, adults, men, women, veterans |
| Thinky: Mindful Journal | IOS | 0 | 4.9 | 59 | E | Focus not depression | Focus on mindfulness without depression focus |
| Thought Challenger | AND | 100 | 5 | 5 | E | Focus not depression | Focus on anxiety and mood not depression |
| Thought Diary Pro | IOS | 0 | 3.6 | 9 | I | - |  |
| Three Good Things | IOS | 0 | 4.9 | 32 | E | Focus not depression | Journaling with no focus on depression |
| Three Good Things - A Happiness Journal | IOS | 0 | 4.8 | 1004 | E | Focus not depression | Journaling with no focus on depression |
| Thrive | NHS |  |  |  | E | Focus not depression | Focus on anxiety and stress |
| Togetherall | NHS | 0 | 4.8 | 1004 | E | No access | Teens, parents, adults, men, women, veterans |
| tomo | IOS | 0 | 4.7 | 11 | E | Focus not depression | Habit building |
| Treatment of depression with the Qur'an | AND | 500 | NA | NA | E | No active component | Religious quotes and images |
| Trill Project | IOS | 0 | 4.5 | 1212 | E | Focus not depression | Thought diary |
| Ultima Fitness & Wellness | IOS | 0 | 5 | 22 | E | Focus not depression | Fitness and wellness app |
| Unhappy Wallpapers - Atlas Labs | AND | 0 | 4.2 | 26 | E | No active component | Providing quotes |
| Unhappy wallpapers - Leafgreen | IOS | 1000000 | 4.2 | 3974 | E | No active component | Providing quotes |
| Unhappy wallpapers 😞 | AND | 10000 | 4.3 | 66 | E | No active component | Providing quotes |
| Unique Daily Affirmations | IOS | 0 | 4.7 | 15911 | E | No active component | Providing quotes |
| Untire: Beating cancer fatigue | NHS |  |  |  | E | Focus not depression | Cancer fatigue |
| Unwinding Anxiety | IOS | 0 | 4.9 | 936 | E | Focus not depression | Focus on anxiety |
| UP! - Depression, bipolar & borderline management | AND | 50000 | 4.3 | 1975 | I | - |  |
| UpLift - Depression & Anxiety | IOS | 0 | 4.8 | 52 | I | - |  |
| Uplifter - Positive Psychology, Gratitude Journal, Mood Tracker | IOS | 0 | 3.7 | 7 | E | Focus not depression | Focus on positive psychology not depression |
| vCreate | NHS |  |  |  | E | Focus not depression | Video Consultation |
| Vent - Express yourself freely | AND | 1000000 | 3.8 | 14078 | E | Focus not depression | Social diary |
| Vibrator | IOS | 0 | 4 | 512 | E | Focus not depression | Phone vibration for meditation |
| WaitLess | NHS |  |  |  | E | Focus not depression | Scheduling and information tool from the NHS |
| Wallpapers For Depression | IOS/AND | 10 | NA | NA | E | No active component | Providing quotes |
| Wana: We are not alone | IOS | 0 | 4 | 62 | E | Focus not depression | Network for "Lyme disease, chronic fatigue syndrome, fibromyalgia, Crohn’s disease, Ehlers-Danlos syndrome, endometriosis, and POTS" |
| War On Cancer - Social App | IOS | 0 | 4.9 | 14 | E | Focus not depression | Cancer |
| Waver: Anxiety & Depression | IOS | 0 | NA | NA | E | No access | No access |
| WellMind | AND | 10000 | 3.3 | 105 | I | - |  |
| Wellness Hub: Video Counseling app for Depression | IOS/AND | 10000 | 4.3 | 144 | I | - |  |
| Wellness: Mood Meds & Health | IOS | 0 | 4.4 | 256 | E | Focus not depression | Focus on wellness |
| WellTrack - Interactive Self-Help Therapy | IOS/AND | 10000 | 3.1 | 58 | I | - |  |
| Welzen - Meditation app calm | IOS | 0 | 4.5 | 73 | E | Focus not depression | Focus on meditation without depression focus |
| What's My M3 | ADAA | 0 | 0 | 0 | E | No access | No access |
| What's up? - A mental health app | IOS/AND | 500000 | 4.3 | 3183 | I | - |  |
| Whil: Mindfulness and Meditation | IOS | 0 | 4.8 | 2459 | E | Focus not depression | Focus on meditation without depression focus |
| Whispers from God - Christian Meditation | AND | 10000 | 4.7 | 208 | E | No active component | Religious quotes and images |
| Wholesome World | NHS |  |  |  | E | Focus not depression | Teens, parents, adults, men, women, veterans |
| Wim Hof Method | IOS | 0 | 4.9 | 1888 | E | Focus not depression | Breathing training |
| Wisdo | IOS | 0 | 4.5 | 8663 | E | Focus not depression | Network for physical and mental wellness and motivation |
| Woebot: Your Self-Care Expert | AND | 100000 | 4.5 | 7103 | I | - |  |
| Worry Knot | AND | 100 | 5 | 3 | E | Focus not depression | Decrease emotional responses |
| Worrydolls | IOS | 0 | 4.8 | 176 | E | Focus not depression | Focus on anxiety |
| WorryTree | NHS |  |  |  | E | Focus not depression | Focus on anxiety |
| WorryWatch | ADAA | 0 | 4.8 | 176 | E | Focus not depression | Focus on anxiety |
| Wysa: stress, depression & anxiety therapy chatbot | IOS/AND | 1000000 | 4.7 | 45848 | I | - |  |
| Yellow Card | NHS |  |  |  | E | Focus not depression | Reporting suspected adverse reactions to all medicines |
| Yoga for Depression | AND | 100 | NA | NA | I | - |  |
| Yoga for Relief of Anxiety, Stress and Depression | AND | 10000 | 4.3 | 86 | I | - |  |
| You Are Important - Depression, Suicide, & Bullying Prevention Videos App by Wonderiffic® | IOS | 0 | 3.8 | 5 | E | No access | No access |
| Youper - Emotional Health | IOS/AND | 1000000 | 4.6 | 47262 | I | - |  |
| Zen Lounge: Meditation Sounds | IOS | 0 | 4.7 | 46 | E | Focus not depression | Focus on meditation without depression focus |
| Zen meditation relax sound | IOS | 0 | 4.7 | 29 | E | Focus not depression | Focus on meditation without depression focus |
| Zulu - Logical Puzzle Game | IOS | 0 | 4.4 | 167 | E | Focus not depression | Game |

**Table S8.** List of reasons for rating an app to have no active components, occurrences of this reason and further explanation why this specific reason was not considered an active component.

| **Reason for Exclusion** | **Occurrences** | **Explanation** |
| --- | --- | --- |
| Providing quotes | 49 | Quotes and text snippets embedded in pictures or illustrations are presented without any further interaction. |
| Depression triage without any intervention components | 35 | App based form of a validated or not validated screening instruments. |
| Information about depression | 13 | Information about depression presented in an article format without the goal of eliciting behavior change or engaging with individuals. |
| Religious quotes and images | 3 | Religious quotes and text snippets embedded in pictures or illustrations are presented without any further interaction. |
| Information from social media posts | 1 | Quotes and text snipes derived from social media posts without the goal to elicit behavior. |
| Food information for a range of mental health problems | 1 | Information about food related to mental health (such as depression) presented in an article format without the goal of eliciting behavior change or engaging with individuals. |
| Collection of games with no therapeutic content | 1 | Collection of free-time games without any therapeutic or behavior change focus. |
| Community plattform to interact without intervention components | 1 | Providing a platform with interaction without presenting intervention components. |
| Tele-therapy video-consultation | 1 | Enabling a video conference with a therapist. No intervention components presented in the app itself. |
| Patients community plattform | 1 | Providing a platform with interaction without presenting intervention components. |

**Table S9.** Calculation of downloads represented by the download category covered by the 18 included apps with more than 500*,*000 downloads in relation to all 118 included apps found on the Apple App and Google Play Store and apps found only on the Google Play Store*.* Calculation taken from Baumel et al. [41].

| **Download Category** | **Apps identified, n** | **Minimum identified app downloads within this category^a^, n** | **Cumulative frequency of app downloads based on download category threshold^b^, n** | **Added percentage of downloads to the overall^c^, %** |
| --- | --- | --- | --- | --- |
| ≥10,000,000 | 3 | 30,000,000 | 30,000,000 | 100.00 |
| 1,000,000 -9,999,999 | 7 | 7,000,000 | 37,000,000 | 18.92 |
| 500,000 - 999,999 | 8 | 4,000,000 | 41,000,000 | 9.76^d^ |
| 100,000 – 499,999 | 8 | 800,000 | 41,800,000 | 1.91 |
| 50,000 – 99,999 | 7 | 350,000 | 42,150,000 | 0.83 |
| 10,000 -49,999 | 20 | 200,000 | 42,350,000 | 0.47 |
| 5000 – 9999 | 6 | 30,000 | 42,380,000 | 0.07 |
| 1000 – 4999 | 13 | 13,000 | 42,393,000 | 0.03 |
| 500 – 999 | 2 | 1000 | 42,394,000 | 0.00 |
| 100 – 499 | 7 | 700 | 42,394,700 | 0.00 |
| 50 – 99 | 4 | 200 | 42,394,900 | 0.00 |
| 10 – 49 | 3 | 30 | 42,394,930 | 0.00 |
| 1 - 9 | 30 | 30 | 42,394,960 | 0.00 |

^a^The number of apps multiplied by the minimum number of downloads based on the download category.

^b^The accumulated number of app downloads in all download categories above including the current download category.

^c^The added percentage of downloads to the total sample if the current download category is added to the review. This percentage is calculated by dividing the total app downloads in this category by the accumulated number of app downloads based on the current category threshold.

^d^The inclusion of apps in the 100,000+ download category would lead to an 1.91% increase of the total number of minimal number of downloads. When the remaining categories would have been included this would have resulted in an increase of 3% of downloads to the total sample.

**Table S10.** Calculation of reviews covered by the five most reviewed apps on the Apple App Store in Relation to all included 35 apps from the Apple App Store. Calculation adapted from Baumel et al. [41].

| **Number of Review** | **Apps identified, n** | **Total number of reviews in this review category^a^, n** | **Cumulative frequency of app reviews based on review category^b^, n** | **Added percentage of reviews to the overall^c^, %** |
| --- | --- | --- | --- | --- |
| 872025 | 1 | 872025 | 872025 | 100.00 |
| 292548 | 1 | 292548 | 1164573 | 25.12 |
| 248387 | 1 | 248387 | 1412960 | 17.58 |
| 47262 | 1 | 47262 | 1460222 | 3.24 |
| 45848 | 1 | 45848 | 1506070 | 3.04 |
| 30240 | 1 | 30240 | 1536310 | 1.97 |
| 27797 | 1 | 27797 | 1564107 | 1.78 |
| 17021 | 1 | 17021 | 1581128 | 1.08 |
| 15771 | 1 | 15771 | 1596899 | 0.99 |
| 15129 | 1 | 15129 | 1612028 | 0.94 |
| 9717 | 1 | 9717 | 1621745 | 0.60 |
| 9302 | 1 | 9302 | 1631047 | 0.57 |
| 7497 | 1 | 7497 | 1638544 | 0.46 |
| 7103 | 1 | 7103 | 1645647 | 0.43 |
| 7071 | 1 | 7071 | 1652718 | 0.43 |
| 5965 | 1 | 5965 | 1658683 | 0.36 |
| 4654 | 1 | 4654 | 1663337 | 0.28 |
| 4643 | 1 | 4643 | 1667980 | 0.28 |
| 4085 | 1 | 4085 | 1672065 | 0.24 |
| 3802 | 1 | 3802 | 1675867 | 0.23 |
| 3710 | 1 | 3710 | 1679577 | 0.22 |
| 3183 | 1 | 3183 | 1682760 | 0.19 |
| 2479 | 1 | 2479 | 1685239 | 0.15 |
| 2208 | 1 | 2208 | 1687447 | 0.13 |
| 1975 | 1 | 1975 | 1689422 | 0.12 |
| 1784 | 1 | 1784 | 1691206 | 0.11 |
| 1660 | 1 | 1660 | 1692866 | 0.10 |
| 1469 | 1 | 1469 | 1694335 | 0.09 |
| 1448 | 1 | 1448 | 1695783 | 0.09 |
| 1229 | 1 | 1229 | 1697012 | 0.07 |
| 1217 | 1 | 1217 | 1698229 | 0.07 |
| 1169 | 1 | 1169 | 1699398 | 0.07 |
| 870 | 1 | 870 | 1700268 | 0.05 |
| 821 | 1 | 821 | 1701089 | 0.05 |
| 602 | 1 | 602 | 1701691 | 0.04 |
| 561 | 1 | 561 | 1702252 | 0.03 |
| 536 | 1 | 536 | 1702788 | 0.03 |
| 460 | 1 | 460 | 1703248 | 0.03 |
| 425 | 1 | 425 | 1703673 | 0.02 |
| 381 | 1 | 381 | 1704054 | 0.02 |
| 380 | 1 | 380 | 1704434 | 0.02 |
| 370 | 1 | 370 | 1704804 | 0.02 |
| 360 | 1 | 360 | 1705164 | 0.02 |
| 318 | 1 | 318 | 1705482 | 0.02 |
| 305 | 1 | 305 | 1705787 | 0.02 |
| 301 | 1 | 301 | 1706088 | 0.02 |
| 290 | 1 | 290 | 1706378 | 0.02 |
| 282 | 1 | 282 | 1706660 | 0.02 |
| 239 | 1 | 239 | 1706899 | 0.01 |
| 224 | 1 | 224 | 1707123 | 0.01 |
| 189 | 1 | 189 | 1707312 | 0.01 |
| 181 | 1 | 181 | 1707493 | 0.01 |
| 178 | 1 | 178 | 1707671 | 0.01 |
| 176 | 1 | 176 | 1707847 | 0.01 |
| 144 | 1 | 144 | 1707991 | 0.01 |
| 137 | 1 | 137 | 1708128 | 0.01 |
| 132 | 1 | 132 | 1708260 | 0.01 |
| 123 | 1 | 123 | 1708383 | 0.01 |
| 117 | 1 | 117 | 1708500 | 0.01 |
| 112 | 1 | 112 | 1708612 | 0.01 |
| 111 | 1 | 111 | 1708723 | 0.01 |
| 105 | 1 | 105 | 1708828 | 0.01 |
| 98 | 1 | 98 | 1708926 | 0.01 |
| 97 | 1 | 97 | 1709023 | 0.01 |
| 86 | 1 | 86 | 1709109 | 0.01 |
| 80 | 1 | 80 | 1709189 | 0.00 |
| 69 | 1 | 69 | 1709258 | 0.00 |
| 58 | 1 | 58 | 1709316 | 0.00 |
| 45 | 1 | 45 | 1709361 | 0.00 |
| 43 | 1 | 43 | 1709404 | 0.00 |
| 41 | 1 | 41 | 1709445 | 0.00 |
| 34 | 2 | 68 | 1709513 | 0.00 |
| 34 | 2 | 68 | 1709581 | 0.00 |
| 27 | 2 | 54 | 1709635 | 0.00 |
| 23 | 1 | 23 | 1709658 | 0.00 |
| 18 | 1 | 18 | 1709676 | 0.00 |
| 17 | 1 | 17 | 1709693 | 0.00 |
| 15 | 1 | 15 | 1709708 | 0.00 |
| 13 | 1 | 13 | 1709721 | 0.00 |
| 12 | 1 | 12 | 1709733 | 0.00 |
| 9 | 2 | 18 | 1709751 | 0.00 |
| 8 | 1 | 8 | 1709759 | 0.00 |
| 6 | 2 | 12 | 1709771 | 0.00 |
| 5 | 1 | 5 | 1709776 | 0.00 |
| 2 | 1 | 2 | 1709778 | 0.00 |
| 1 | 2 | 2 | 1709780 | 0.00 |
| 0 | 28 | 0 | 1709780 | 0.00 |

^a^The number of apps multiplied by the number of reviews based on the review category.

^b^The accumulated number of reviews in all number of review categories above including the current number of review category.

^c^The added percentage of downloads to the total sample if the current number of download category is added to the review. This percentage is calculated by dividing the total number of reviews in this category by the accumulated number of app reviews based on the current category threshold.

^d^The inclusion of apps in the sixth most reviewed app category would lead to an 1.97% increase of the total number of reviews. When the remaining categories would have been included this would have resulted in an increase of 9% of reviews to the total sample.

**Table S11.** Number of studies and type of studies found for each app.

| **App** | **Studies** | **Number Participants** | **Number of Randomized controlled trials** | **Number of Effectiveness Studies** | **Number of Efficacy Studies** | **Studies Comparing App to Other Treatment** |
| --- | --- | --- | --- | --- | --- | --- |
| Calm | 10 | 24,332 | 1 | 1 | 3 | - |
| Headspace | 28 | 3,871 | 15 | 14 | 6 | 3 |
| Daylio | 3 | 14 | - | - | 1 | - |
| Youper | - | - | - | - | - | - |
| Moodpath | 1 | 6,675 | - | - | - | - |
| Wysa | 3 | 129 | - | 1 | - | - |
| Friend Shoulder | - | - | - | - | - | - |
| BetterHelp | 1 | 318 | - | 1 | - | - |
| Sanvello | 2 | 538 | 1 | 2 | - | 1 |
| 7 Cups | 4 | 909 | - | 1 | - | 1 |
| Control and Monitor | - | - | - | - | - | - |
| #SelfCare | - | - | - | - | - | - |
| Remente | - | - | - | - | - | - |
| Reflexio | - | - | - | - | - | - |
| Moodnotes | - | - | - | - | - | - |
| Online therapy […] | - | - | - | - | - | - |
| InnerHour | 1 | - | - | - | - | - |
| Happify | 6 | 155,352 | 1 | 3 | 1 | - |
| What's Up? […] | - | - | - | - | - | - |
| MoodTools […] | 1 | 26 | - | - | - | - |
| DBT Coach | 2 | 38 | - | - | - | - |
| CBT Thought Diary | - | - | - | - | - | - |
| T2 Mood Tracker | 3 | 215 | - | - | - | - |
| Joyable | 1 | 3384 | - | 1 | - | - |
| Breeze | - | - | - | - | - | - |
| Moodkit | 2 | 278 | 2 | - | 2 | 1 |
| Catch It | 2 | 285 | - | 1 | - | - |
| Feeling Good | - | - | - | - | - | - |
| Sum | 70 | 196,364 | 20 | 23 | 14 | 5 |
| Count | 16 | 15 | 5 | 9 | 5 | 4 |
| Mean | 4.25 | 13,090.93 | 4 | 2.56 | 2.8 | 1.25 |
| STD | 6.74 | 39,846.25 | 6.14 | 3.61 | 2.49 | 0.5 |
| Min | 1 | 14 | 1 | 1 | 1 | 1 |
| Max | 28 | 155,352 | 15 | 12 | 7 | 2 |
| Median | 2 | 318 | 1 | 1 | 2 | 1 |
| IQR | 2.25 | 3,455.50 | 1 | 1 | 2 | 0.25 |

**Table S12.** List of the reviewed publications.

| **App** | **Study** | **N** | **Type of Study** | **Focus** | **Comparison to Treatment** |
| --- | --- | --- | --- | --- | --- |
| Calm | Huberty et al (2019)[50] | 88 | Randomized control trial | Efficacy | No |
| Calm | Huberty et al (2019)[51] | 128 | Parallel group | Feasibility | No |
| Calm | Huberty et al (2019)[52] | 128 | Parallel group | Qualitative evaluation | No |
| Calm | Huberty et al (2019)[53] | 12,151 | One-time assessment | Demographics, clinical characteristics, and usage patterns | No |
| Calm | Huberty et al (2020)[54] | 82 | One-time assessment | Qualitative evaluation | No |
| Calm | Puzia et al (2020)[55] | 80 | Parallel group | Efficacy | Compared to psychoeducation |
| Calm | Huberty et al (2020)[56] | 9868 | Cross-sectional study | Qualitative evaluation | No |
| Calm | Clarke et al (2020)[57] | 269 | Pre-post-test, no control group | Efficacy | No |
| Calm | Callender et al (2019)[58] | 1 | A-B single case research design | Effectiveness | No |
| Calm | Puzia et al (2020)[59] | 1537 | One-time assessment | Engagement | No |
| Headspace | Howells et al (2016)[60] | 121 | Randomized control trial | Effectiveness | Compared to other apps |
| Headspace | Lim et al (2015)[61] | 69 | Randomized experimental study | Efficacy | No |
| Headspace | Taylor et al (2016)[62] | 33 | Pre-post-test, no control group | Qualitative evaluation | No |
| Headspace | Laurie et al (2016)[63] | 16 | Qualitative semi-structured interview | Qualitative evaluation | No |
| Headspace | Wen et al (2017)[64] | 43 | Pre-post-test, no control group | Effectiveness | No |
| Headspace | Wylde et al (2017)[65] | 95 | Pre-post-test, no control group | Effectiveness | Compared to intervention |
| Headspace | Mistler et al (2017)[66] | 13 | Pre-post-test, no control group | Feasibility | No |
| Headspace | Bennike et al (2017)[67] | 137 | Randomized control trial | Efficacy | Compared to other apps |
| Headspace | Kubo et al (2018)[68] | 28 | Pre-post-test, no control group | Feasibility | No |
| Headspace | Noone et al (2018)[69] | 91 | Randomized control trial | Effectiveness | Compared to sham meditation |
| Headspace | Yang et al (2018)[70] | 88 | Randomized control trial | Effectiveness | Compared to waitlist |
| Headspace | Economides et al (2018)[71] | 160 | Randomized control trial | Effectiveness | No |
| Headspace | DeSteno et al (2018)[72] | 77 | Randomized experimental study | Efficacy | No |
| Headspace | Rosen et al (2018)[73] | 112 | Randomized control trial | Efficacy | Compared to waitlist |
| Headspace | Champion et al (2018)[74] | 74 | Randomized control trial | Effectiveness | Compared to waitlist |
| Headspace | Kubo et al (2019)[75] | 128 | Randomized control trial | Efficacy | Compared to treatment as usual |
| Headspace | Flett et al (2020)[76] | 250 | Randomized control trial | Effectiveness | Compared to waitlist |
| Headspace | Collins et al (2019)[77] | 65 | Randomized experimental study | Efficacy | No |
| Headspace | Bostock et al (2019)[80] | 238 | Randomized control trial | Effectiveness | Compared to waitlist |
| Headspace | Flett et al (2019)[119] | 208 | Randomized control trial | Effectiveness | Compared to other apps |
| Headspace | Kirk et al (2019)[79] | 77 | Randomized experimental study | Efficacy | Compared to other apps and control |
| Headspace | Quinones et al (2019)[80] | 994 | Randomized control trial | Effectiveness | Compared to other apps and control |
| Headspace | Björkstrand et al (2019)[81] | 29 | Randomized control trial | Effectiveness | Compared to waitlist |
| Headspace | Flett et al (2019)[82] | 174 | Randomized control trial | Adherence | No |
| Headspace | Avalos et al (2020)[83] | 16 | Pre-post-test, no control group, no randomization | Feasibility | No |
| Headspace | Nübold et al (2020)[84] | 209 | Multi-source field study | Gains in leadership | No |
| Headspace | Rung et al (2020)[85] | 236 | Pre-post-test, no control group | Feasibility | No |
| Headspace | Ball et al (2020)[86] | 90 | Randomized control trial | Qualitative evaluation | Compared to treatment as usual |
| Daylio | Hussain et al (2020)[87] | 14 | Lab experiment, no randomization | Efficacy | No |
| Daylio | Cristol et al (2018)[88] | - | Viewpoint | Patients Perspective | No |
| Daylio | Chaudhry et al (2016)[89] | - | Viewpoint | App's characteristics | No |
| Moodpath | Scherr et al (2019)[90] | 6675 | Study, no randomization, no control group | Health-seeking behavior | No |
| Wysa | Inkster et al (2018)[91] | 129 | Pre-post-test, no control group | Effectiveness | No |
| Wysa | Kretzschmar et al (2019)[92] | - | Viewpoint | Ethical Perspective | No |
| Wysa | Inkster et al (2020)[93] | - | Viewpoint | Feasibility | No |
| BetterHelp | Marcelle et al (2019)[94] | 318 | Pre-post-test, no control group | Effectiveness | No |
| Sanvello | Moberg et al (2019)[95] | 500 | Randomized control trial | Effectiveness | Compared to waitlist |
| Sanvello | Broglia et al (2019)[96] | 38 | Two-arm, parallel, nonrandomized | Effectiveness | Compared to receiving face-to-face treatment |
| 7 Cups | Baumel et al (2015)[97] | 866 | One-time assessment | Qualitative evaluation | Compared to satisfaction in psychotherapy |
| 7 Cups | Baumel et al (2016)[98] | 14 | One-time assessment | Qualitative evaluation | No |
| 7 Cups | Baumel et al (2018)[99] | 19 | Pre-post-test, no control group, no randomization | Effectiveness | No |
| 7 Cups | Baumel et al (2016)[100] | 10 | One-time assessment | Overall quality of app and listeners' knowledge and confidence | No |
| InnerHour | Malik et al (2020)[101] | - | Viewpoint | Feasibility | No |
| Happify | Carpenter et al (2016)[102] | 152,747 | Pre-post-test, no control group, no randomization | Effectiveness | No |
| Happify | Parks et al (2018)[103] | 1053 | Randomized control trial | Effectiveness | Compared to psychoeducation |
| Happify | Williams et al (2018)[104] | 591 | Parallel group | Resilience | Compared to psychoeducation |
| Happify | Hunter et al (2019)[105] | 140 | Randomized experimental study | Efficacy | Compared to psychoeducation |
| Happify | Martinez-Guzman et al. 2019 [106] | - | Viewpoint | Ethical Perspective | No |
| Happify | Parls et al (2020)[107] | 821 | Pre-post-test, no control group, no randomization | Effectiveness | No |
| MoodTools | Sarkar et al (2016)[108] | 26 | Lab experiment, no randomization | Usability | Compared to psychoeducation |
| DBT Coach | Rizvi (2011)[109] | 22 | Pre-post-test, no control group, no randomization | Feasibility | No |
| DBT Coach | Rizvi (2016)[110] | 16 | Pre-post-test, no control group, no randomization | Feasibility, acceptability, usability, and immediate effects | No, but treatment was offered as an adjunction to 6-month dialectical behavior therapy |
| T2 Mood Tracker | Bush (2014)[111] | 8 | One-time assessment | Feasibility | No |
| T2 Mood Tracker | Dewar (2016)[112] | 191 | Randomized experimental study | Reliability of a measure for motivation | No |
| T2 Mood Tracker | McCreight et al (2019)[113] | 16 | Evaluation using participatory design methods | Usability | No |
| Joyable | Dryman et al (2017)[114] | 3384 | Pre-post-test, no control group, no randomization | Effectiveness | No |
| Moodkit | Bakker et al (2018)[115] | 226 | Randomized control trial | Efficacy | Compared to waitlist |
| Moodkit | Dahne et al (2019)[116] | 52 | Randomized control trial | Efficacy | Compared to treatment as usual |
| Catch It | Kindermann et al (2016)[117] | 285 | Pre-post-test, no control group, no randomization | Effectiveness | No |
| Catch It | Jayachandran CM et al (2017) [118] | - | Conception | Algorithm Development | No |
| Sum | - | 196,364 | - | - | - |
| Count | - | 63 | - | - | - |
| Mean | - | 3,116.89 | - | - | - |
| STD | - | 19,118.68 | - | - | - |
| Min | - | 1 | - | - | - |
| Max | - | 15,2747 | - | - | - |
| Median | - | 112 | - | - | - |
| IQR | - | 208.5 | - | - | - |

Comments: Mean, STD, Median, IQR calculated over count of all 68 identified studies.
